# Supplementary material for: Single-cell analysis of lizard blastema fibroblasts reveals phagocyte-dependent activation of Hedgehog-responsive chondrogenesis
Source: Nat Commun. 2023 Aug 10;14:4489. doi: 10.1038/s41467-023-40206-z (PMC10415409; doi:10.1038/s41467-023-40206-z)
Supplement: Supplementary file 1 — Supplementary Information [file 41467_2023_40206_MOESM1_ESM.pdf]

Supplementary Information for

Single-cell analysis of lizard blastema fibroblasts reveals phagocyte-dependent activation of Hedgehog-responsive chondrogenesis

Ariel C Vonk, Xiaofan Zhao, Zheyu Pan, Megan L Hudnall, Conrad G Oakes, Gabriela A Lopez, Sarah C Hasel-Kolossa, Alexander W C Kuncz, Sasha B Sengelmann, Darian J Gamble, Thomas P Lozito*

***Correspondence**: Thomas P Lozito
Email: lozito@usc.edu

This PDF file includes:

Supplementary Methods
Supplementary Figures 1-23
Supplementary Tables 1-3
Supplementary References

SUPPLEMENTARY METHODS

Immunofluorescence (IF) staining detailed protocols

Col2 IF

Slides were rinsed in phosphate-buffered saline (PBS) three times to remove Optimal Cutting Temperature compound (OCT) embedding medium, then equilibrated in 0.02% bovine serum albumin (BSA) in PBS for 15 minutes at room temperature. Samples were then washed once more with 0.02% BSA/PBS and incubated with Chondroitinase ABC (100 mU/mL, Sigma Aldrich, PN: C3667) and Hyaluronidase (250 U/mL, Sigma Aldrich, PN: H3506) in 0.02% BSA/PBS solution for antigen retrieval for 30 minutes at 37°C. Slides were washed twice in 1X tris-buffered saline (TBS), permeabilized at room temperature in 0.01% Triton X-100/TBS for 10 minutes, then washed twice for 5 minutes each in 0.1% Tween® 20/TBS. Samples were blocked with filtered blocking buffer (1% BSA and 0.1% Tween® 20 in TBS) for 2 hours at room temperature. Primary anti-Col2 antibody (Abcam, PN: ab34712) was diluted 1:1000 in blocking buffer and samples were incubated overnight at 4°C. Slides were washed twice for 5 minutes each with 0.1% Tween® 20/TBS, then anti-Mouse IgG (H+L) cross-adsorbed Alexa Fluor™ 647-tagged secondary antibody (Invitrogen, PN: A-21235), 1:500 dilution in blocking buffer, was added for 1 hour at room temperature in the dark. Slides were again washed twice for 5 minutes each with 0.1% Tween® 20/TBS and equilibrated in TBS for 5 minutes. Slides were counterstained with 300 nM DAPI for 10 minutes, washed twice with TBS and mounted in hard-set VectaMount® mounting media (Vector Laboratories, PN: H-5000). Slides were stored at 4°C prior to imaging.

Ctsk IF

Slides were rinsed in PBS three times to remove OCT embedding medium, then equilibrated in TBS for 15 minutes at room temperature. Samples were then permeabilized at room temperature in 0.01% Triton X-100/TBS for 10 minutes and washed twice for 5 minutes each with 0.1% Tween® 20/TBS. Samples were blocked with filtered blocking buffer (1% BSA and 0.1% Tween® 20 in TBS) for 2 hours at room temperature. Primary anti-Ctsk antibody (Abcam, PN: ab19027) was diluted 1:200 in blocking buffer and samples were incubated overnight at 4°C. Slides were washed twice for 5 minutes each with 0.1% Tween® 20/TBS, then anti-rabbit-IgG (H+L) cross-adsorbed Alexa Fluor™ 647-tagged secondary antibody (Invitrogen, PN: A-21244), 1:500 dilution in blocking buffer, was added for 1 hour at room temperature. Slides were again washed twice for 5 minutes each with 0.1% Tween® 20/TBS and equilibrated in TBS for 5 minutes. Slides were counterstained with 300 nM DAPI for 10 minutes, washed twice with TBS and mounted in hard-set VectaMount® mounting media (Vector Laboratories, PN:H-5000). Slides were stored at 4°C prior to imaging.

Real time-polymerase chain reaction (RT-PCR)

Total RNA of tails collected 14 days post-amputation (DPA) from lizards (*Anolis carolinensis*) treated with vehicle control, Hedgehog (Hh) inhibitor cyclopamine, or Hh smoothened agonist (SAG) were isolated using TRIzol™ reagent (Invitrogen, PN: 15596026) and purified using RNeasy® Plus Mini Kit (Qiagen, PN: 74134). Reverse transcription reactions were performed using Superscript® VILO™ cDNA Synthesis Kit (Invitrogen, PN: 11754250) according to the manufacturer's instructions. RT-PCR was performed for anole *sulf1*, *spp1*, *pltp*, and *sall1* using Applied Biosystems™ SYBR™ Green PCR Master Mix (ThermoFisher Scientific, PN: 4309155) with a StepOnePlus™ RT-PCR thermocycler (Applied Biosystems). All sample values were normalized to *gapdh* using the $2^{-\Delta\Delta Ct}$ method and relative gene expression levels were generated by normalizing to corresponding vehicle control conditions for each gene. Forward and reverse primer sequences are listed in Supplementary Table 4.

Phagocytosis assays

pHrodo™ green bioparticles for phagocytosis

Lizard (*A. carolinensis*) phagocytes were incubated with 0, 1 or 100 µg/mL pHrodo™ green *E. coli* bioparticles (ThermoFisher Scientific, PN: P35366) for 2 hours at 30°C prior to analysis via BD FACSAria™ II flow cytometer. pHrodo™ bioparticles exhibit pH-sensitive fluorescent green signal that substantially increases with lower pH, such as upon ingestion into acidic phagosomes[1], allowing for fluorescent detection of phagocytic cells. Forward scatter and side scatter gating strategies depicted in Supplementary Figure 23.

Fluoroliposome® DiI

Green anole lizards (*A. carolinensis*) and mourning geckos (*Lepidodactylus lugubris*) with regenerated tails 21 DPA were weighed and received 0.125 mg/g animal weight Fluoroliposome® DiI (Encapsula Nano Sciences, PN: CLD-8911) via intraperitoneal injections. Fluoroliposome® DiI is a dye, DiI, packaged in liposomes that fluorescence when phagocytosed and incorporated in lipophilic membranes, allowing for quantification of phagocytic cells *in vivo*[2]. Tails were collected 4-6 hours later for histology and analysis via fluorescence microscopy.

Flow cytometry

Primary antibodies anti-Ctsb (Abcam, PN: ab190077, 1:500 dilution) and anti-Ctsk (Abcam, PN: ab19027, 1:500 dilution) were conjugated to Alexa Fluor™ 488 and 647 fluorophores, respectively, prior to incubation using Zip Alexa Fluor™ 488 and 647 Rapid Antibody Labeling Kits (Invitrogen, PNs: Z11233, Z11235), according to manufacturer's instructions. Enriched macrophage and septoclast cell pools were pelleted and resuspended in ice-cold PBS with 10% fetal calf serum and 1% sodium azide. Suspensions were then incubated, with prepared conjugated primary antibodies anti-Ctsb-Alexa Fluor™ 488 and anti-Ctsk-Alexa Fluor™ 647 in ice-cold PBS with 3% BSA for 30 minutes at 4°C. Cells were washed 3 times with ice-cold PBS via centrifugation at 400 xg for 5 minutes each and resuspended in ice-cold PBS with 3% BSA and 1% sodium azide. Cells were then analyzed with BD FACSAria™ II flow cytometer. Cells were single-gated from total events based on forward scatter and side scatter. Quadrants were then gated based on control macrophage or septoclast sample cell pools incubated with polyclonal rabbit IgG isotype (Abcam, PN: ab171870) with matching fluorophores. Data was analyzed using FlowJo software (FlowJo LLC, version 9). Quadrant gating strategy depicted in Supplementary Figure 19. Forward scatter and side scatter gating strategies depicted in Supplementary Figure 23.

FIJI (Image J) image quantification analysis

IF quantification

To quantify percentages of fluorescently labeled cells within $Col2^+$ regions, fluorescent images were separated into DAPI, Col2, DiI, and/or DiO channels. Over/under thresholding was used to identify positive staining in each channel. The lower-upper limit thresholds on a 0-255 scale were: DiI/DiO 25-85; Col2 45-85; DAPI 25-200. Next, DiI/DiO and DAPI channels were overlaid, and cell nuclei associated with DiI/DiO signal were labeled with ascending numbers counted. Finally, images of labeled nuclei were overlayed onto corresponding Col2 images and labeled nuclei within $Col2^+$ regions were marked with ascending letters and counted. Percentages of fluorescently labeled cells within $Col2^+$ regions were calculated from counts of labeled cells within $Col2^+$ regions and counts of total cells labeled with DiI/DiO.

FISH quantification

To quantify cell densities of phagocytes based on *ctsb*, *ctsk*, and *col4a1* expression, fluorescent microscope images were divided into 1 mm^2 tail/limb regions and separated into DAPI, *ctsb*, *ctsk*, and *col4a1* channels. Over/under thresholding was used to identify positive staining in each channel. The lower-upper limit thresholds on a 0-255 scale were: *ctsb* 50-255; *ctsk* 15-255; *col4a1*; 50-255; DAPI 25-200. Next, DAPI channels were

successively overlaid with *ctsb*, *ctsk*, and *col4a1* channels, and cell nuclei associated with each marker were labeled and counted. Macrophage, osteoclast, and septoclast cell numbers were quantified from counts of *ctsb*$^+$ *ctsk*$^-$ *col4a1*$^-$, *ctsb*$^+$ *ctsk*$^+$ *col4a1*$^-$, *ctsb*$^-$ *ctsk*$^+$ *col4a1*$^+$ cells, respectively. Total sample areas were calculated based on DAPI signal. Finally, cell densities were calculated based on cell counts and sample areas.

To quantify contributions of select cell populations to regenerated tissues, 5 tails phenotypically and morphologically representative of each of the four time points tested (Day 0, Inflammatory Stage, Blastema Stage, Regenerated Homeostasis) were sagittally sectioned. Five middle sections were analyzed by FISH in the Cy5 channel for fibroblastic connective tissue cells, or FCTCs (*col3a1*), phagocytic macrophage and osteoclast (*ctsb*), immune cell (*aoah*), epithelial (*krt5*), muscle-related (*ckm*), chondrocyte (*col2a1*), endothelial (*vwf*), and ependymal (*fabp7*) markers. All sections were counterstained with DAPI. Over/under thresholding was used to identify positive staining in each channel. The lower-upper limit thresholds on a 0-255 scale were: Cell markers 50-255; DAPI 25-200. Total sample and marker signal areas were measured via FIJI/ImageJ from DAPI and Cy5 channels, respectively. Amputation planes were identified by distal most mature scale and tail vertebrae. Total sample areas included all DAPI$^+$ area distal to boundaries 500 µm proximal to amputation planes. Percent areas for each marker were calculated from marker signal areas and corresponding total sample areas.

Gross tail length quantification

To quantify regenerated tail length, the straight-line distance between the amputation plane and distal tail tip was calculated using the FIJI/ImageJ measure tool. Amputation planes were identified by distal most mature scale and tail vertebrae.

SUPPLEMENTARY FIGURES

Supplementary Figure 1. Single-cell RNA sequencing (scRNAseq) cluster gene expression and validation. (a) Percentage and average expression of top differentially expressed genes by cluster of regenerating tail time course scRNAseq dataset presented in Figure 1a. **(b-i)** UMAP feature plots of cluster-defining marker gene expression within regenerating tail scRNAseq dataset. **(j-q)** Representative RNAscope™ *in situ* hybridization (ISH) of cluster-defining marker gene expression in lizard (*Anolis carolinensis*) tails with respective **(r-y)** magnified insets. n = 5 lizards per time point assayed. **(j-q)** Bar = 500 µm; **(r-y)** Bar = 50 µm.

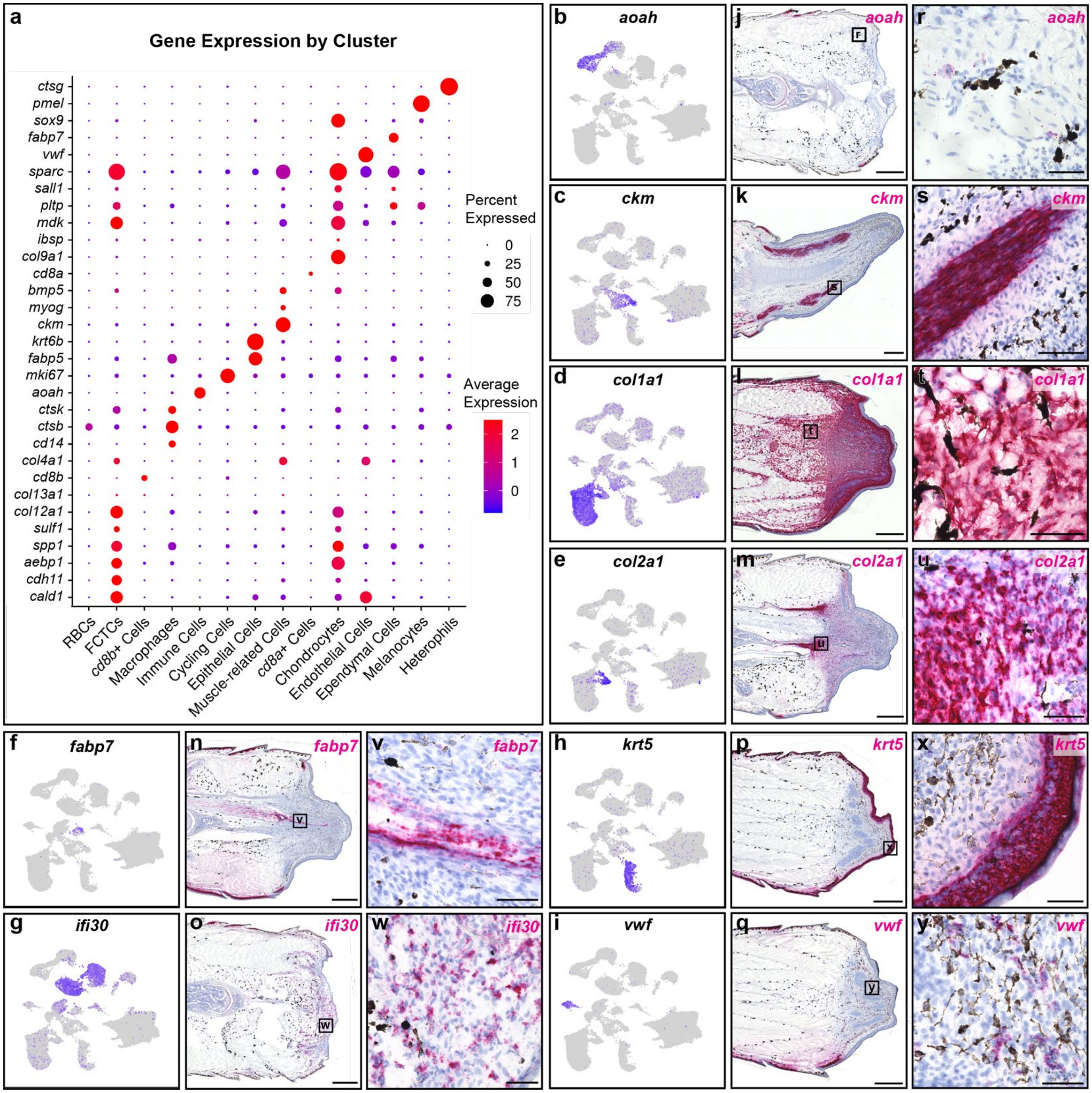

Supplementary Figure 2. Fluorescent ISH quantification of proportional area contributions of respective cell types by scRNAseq cluster. Quantification of positive RNAscope™ fluorescent ISH (FISH) percentage area stained of regenerating tail at day 0, inflammatory stage (7 days post-amputation, or DPA), blastema stage (14 DPA), and regenerated homeostasis (28 DPA), for respective scRNAseq clusters with indicated marker genes: FCTCs (*col3a1*), phagocytic macrophages and osteoclasts (*ctsb*), immune cells (*aoah*), epithelial cells (*krt5*), muscle-related cells (*ckm*), chondrocytes (*col2a1*), endothelial cells (*vwf*), and ependymal cells (*fabp7*). n = 5 tail samples quantified per time point per cell type. One-way Welch's ANOVA for unequal variances with Dunnett's T3 multiple comparisons tests were used per gene between respective tail stages. *, adjusted $p < 0.05$; **, adjusted $p < 0.01$; ***, adjusted $p < 0.001$; ns, not significant**.** Adjusted p-values represent Dunnett's T3 multiple comparisons tests compared to Day 0 within cell types, unless otherwise noted. Data are presented as mean values +/- standard deviation. Source data are provided as a Source Data file.

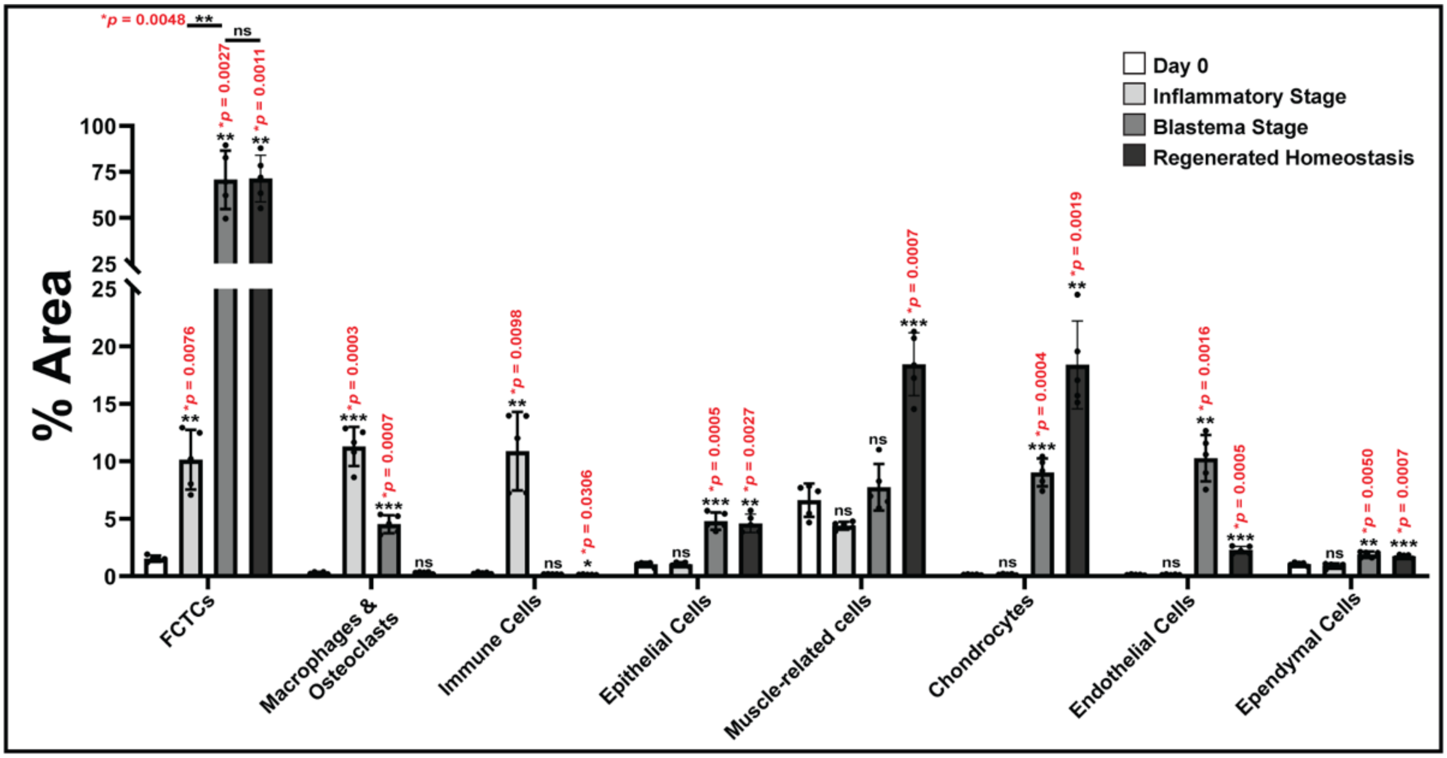

Supplementary Figure 3. FCTC/chondrocyte pseudotime trajectory analysis and TSNE subclustering by tail stage. (a, b) Regeneration stages mapped on **(a)** pseudotime trajectory for FCTC/chondrocyte subset and **(b)** FCTC/chondrocyte subset TSNE. **(c-j)** Pseudotime trajectory and TSNE FCTC/chondrocyte subclustering for **(c, d)** Day 0, **(e, f)** Inflammatory Stage**, (g, h)** Blastema Stage, or **(i, j)** Regenerated Homeostasis stage tail scRNAseq sample cells only.

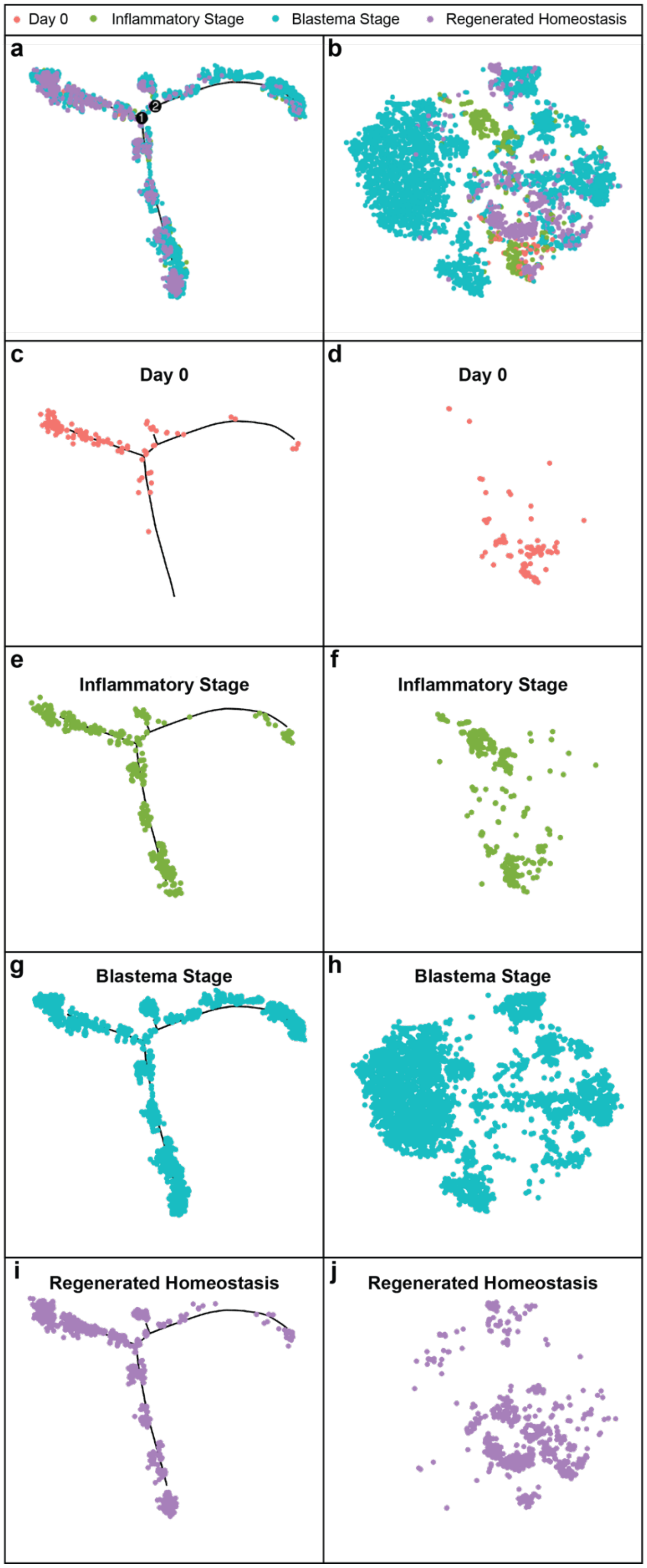

Supplementary Figure 4. FCTC/chondrocyte subcluster gene expression and pseudotime trajectory analysis of *sall1* and *pltp*. (a, b) Gene expression overlaid on TSNE subclustering of FCTC/chondrocyte subset and **(c, d)** pseudotime trajectory analysis for fibroblast marker genes **(a, c)** *pltp* and **(b, d)** *sall1***.**

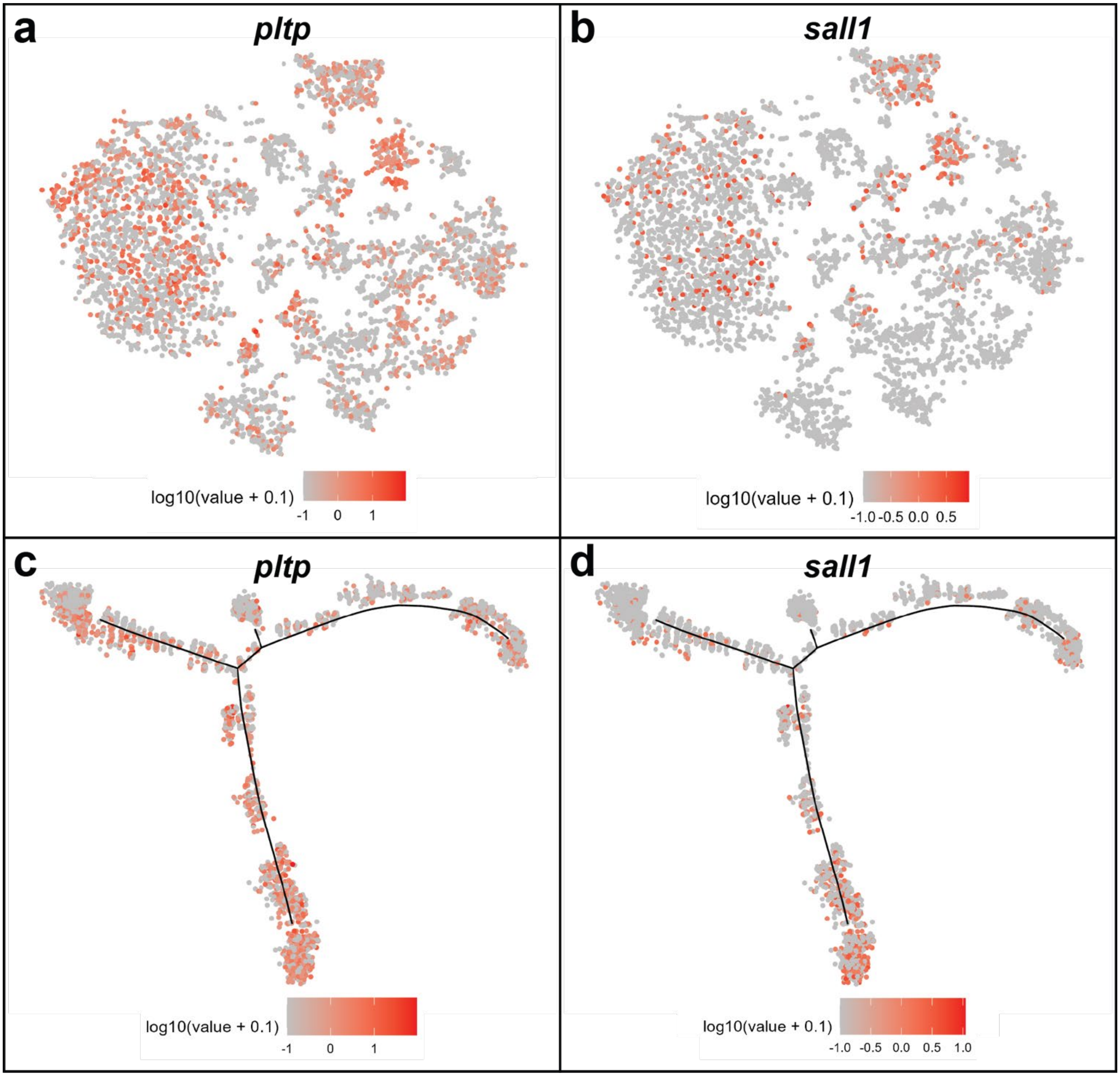

Supplementary Figure 5. Additional fibroblast gene expression throughout lizard tail regeneration. Representative sagittal sections of original and regenerating lizard (*A. carolinensis*) tail samples analyzed via ISH. **(a-e)** Original tails (0 DPA) with select magnified lower right corner insets featuring homeostatic fibroblast expression, **(f-j)** inflammatory stage tails 7 DPA, **(k-o)** blastema stage tails 14 DPA, and **(p-t)** regenerated homeostasis stage tails 28 DPA analyzed for *cdh11, col12a1, mdk, sparc*, and tenascin-like (*tnl*) expression. n = 5 lizards/samples per time point assayed. ct, cartilage tube; et, ependymal tube; rm, regenerated muscle; sc, spinal cord; ve, vertebra. Bar = 500 µm.

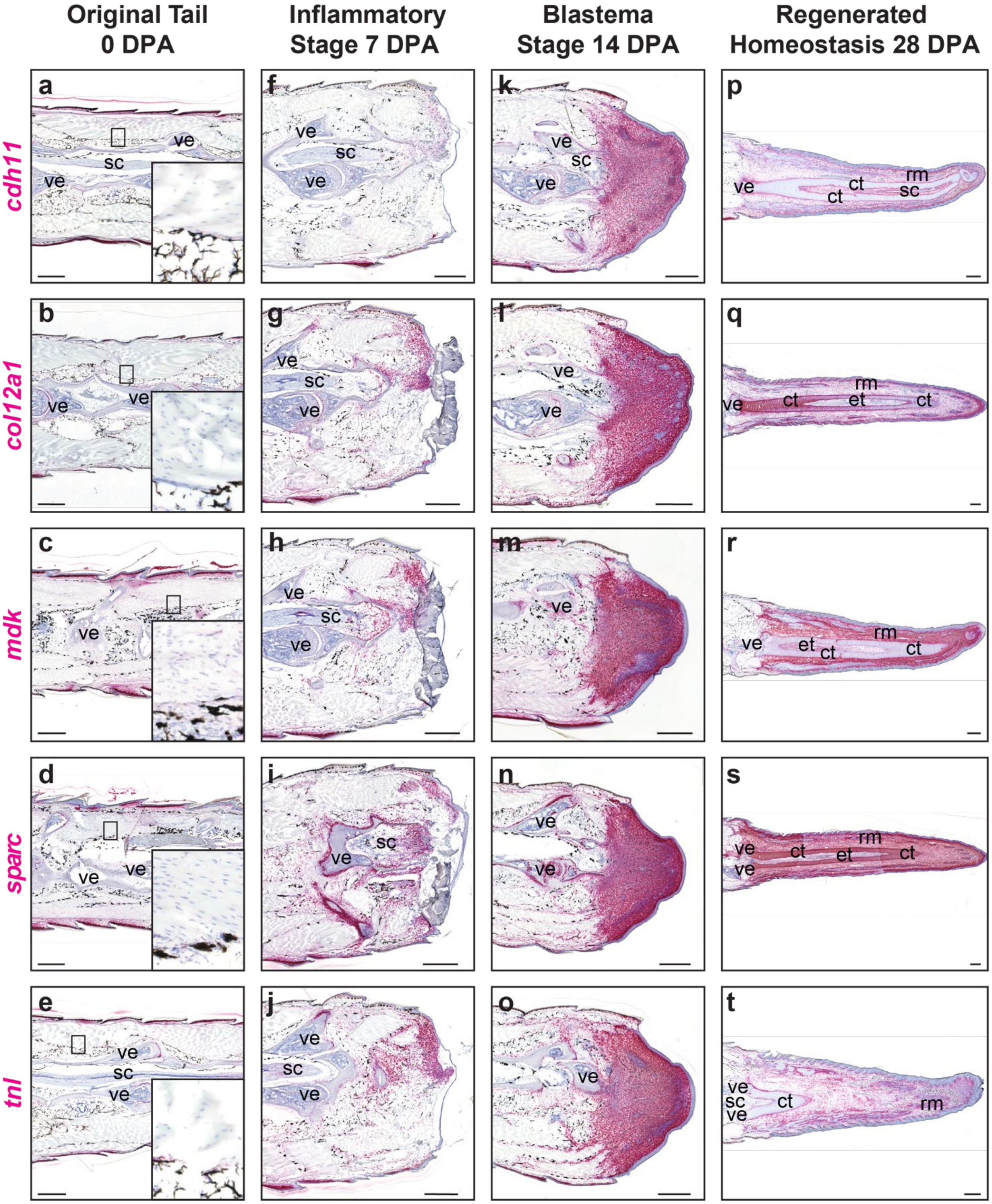

Supplementary Figure 6. Hedgehog signaling regulates blastema cell chondrogenesis. Representative tail samples collected 28 DPA from lizards (*Lepidodactylus lugubris*) treated with vehicle control (PBS), Hedgehog (Hh) pathway inhibitor cyclopamine, or Hh pathway smoothened agonist, SAG, analyzed by **(a-c)** gross morphology and **(d-l)** histology/FISH for *shh* (Hh signal), *col2a1* (cartilage), and *fabp7* (neural stem cells). **(d-f)** Sagittal representative tail sections and **(g, i, k)** corresponding cross sections of regenerated tails. **(h, j, l)** Higher magnification views of ependymal tube cross sections identified in **(g, i, k)**, respectively. n = 5 lizards per treatment condition. ct, cartilage tube; ec, ectopic cartilage; et, ependymal tube. **(a-f)** Bar = 2.5 mm. **(g, i, k)** Bar = 500 µm. **(h, j, l)** Bar = 50 µm.

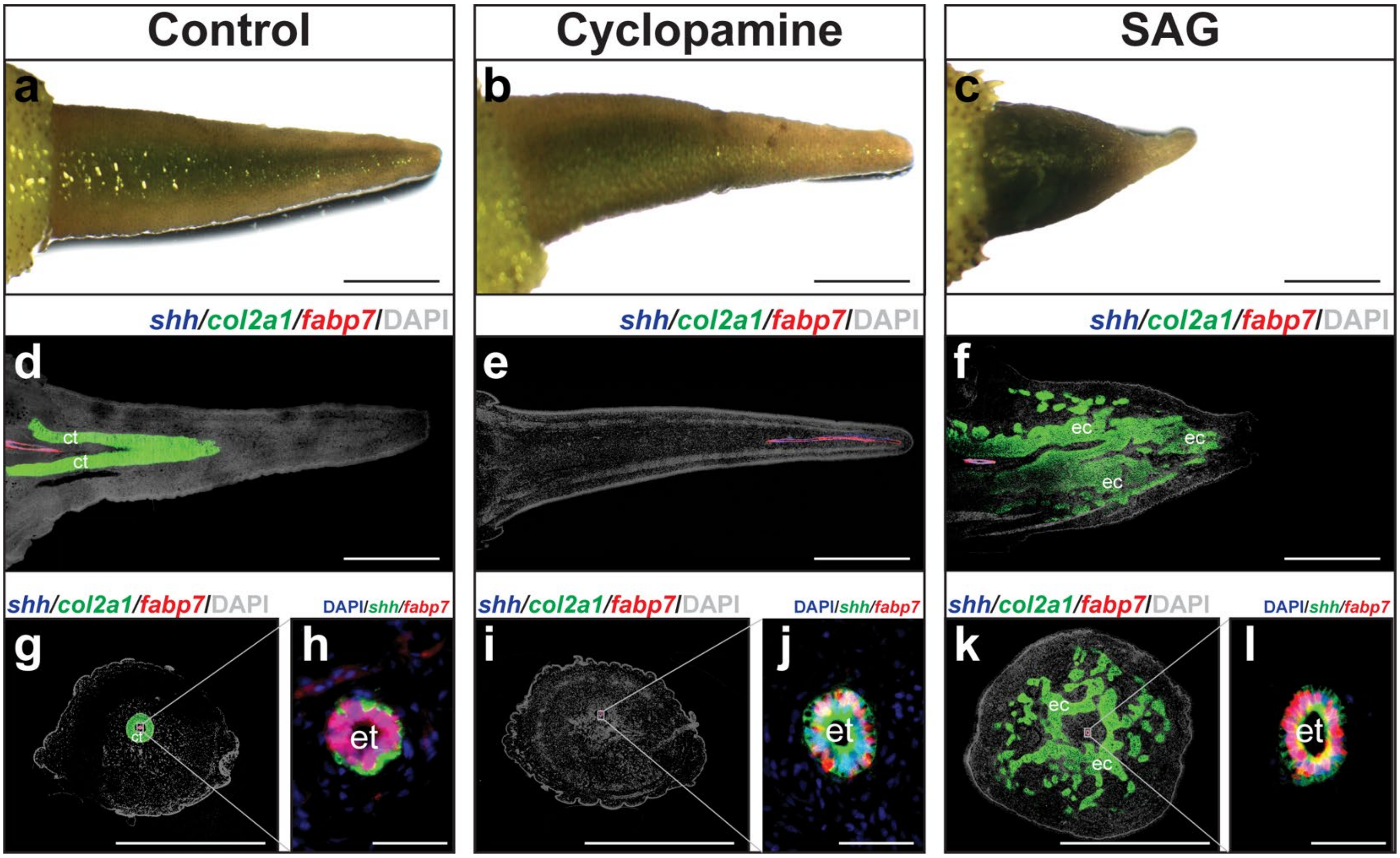

Supplementary Figure 7. Lizard tail, but not limb, fibroblasts undergo Hedgehog-regulated chondrogenesis. Sagittal sections of representative **(a-c, g-i, m-o)** tails and **(d-f, j-l, p-r)** limb samples collected from lizards (*A. carolinensis*) 28 DPA treated with **(a-f)** vehicle control, **(g-l)** cyclopamine, or **(m-r)** SAG, and analyzed by histology/ISH for *col2a1, spp1* and *gli1*. n = 5 lizards per treatment condition. tb, tibia; ve, vertebra. Bar = 500 µm.

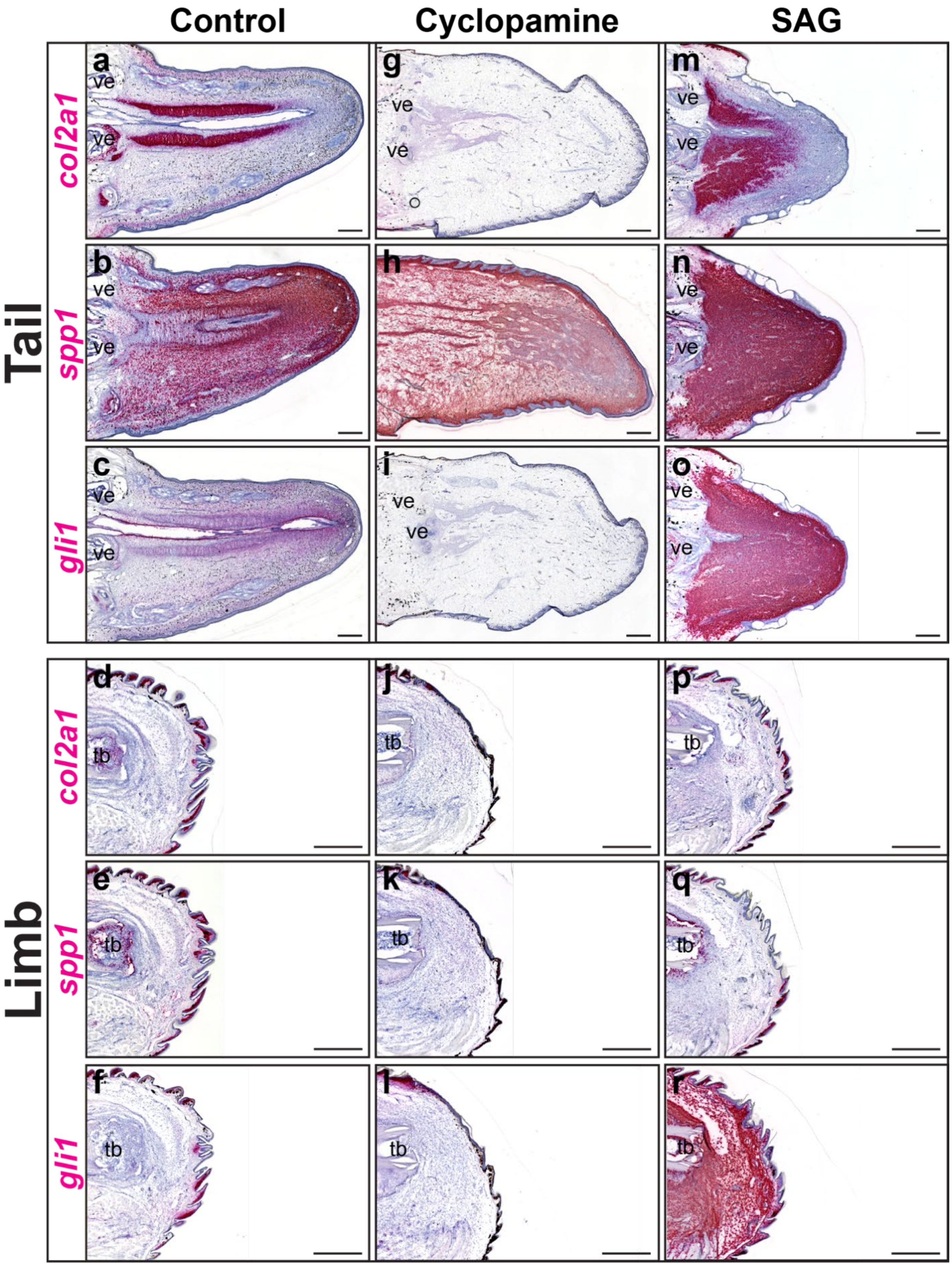

Supplementary Figure 8. Real time-polymerase chain reaction (RT-PCR) reveals *sulf1* expression is Hedgehog signaling-dependent in regenerating lizard tail. RT-PCR analyses of lizard *sulf1*, *spp1*, *pltp*, and *sall1* expression in tails collected 14 DPA from lizards *(A. carolinensis)* treated with vehicle control, cyclopamine, or SAG. All gene expression levels were first normalized to corresponding *gapdh* expression levels to control for possible changes in total mRNA amounts caused by drug treatments. Relative gene expression levels were then generating by normalization to corresponding vehicle control conditions. n = 5 tails per drug treatment condition assessed across 5 RT-PCR runs. Unpaired two-way *t*-tests with Welch's correction for unequal variances were used. *, $p = 0.0366$; ***, $p = 0.0008$; ****, $p < 0.0001$; ns, not significant, compared to corresponding vehicle controls. Data are presented as mean values +/- standard deviation. Source data are provided as a Source Data file.

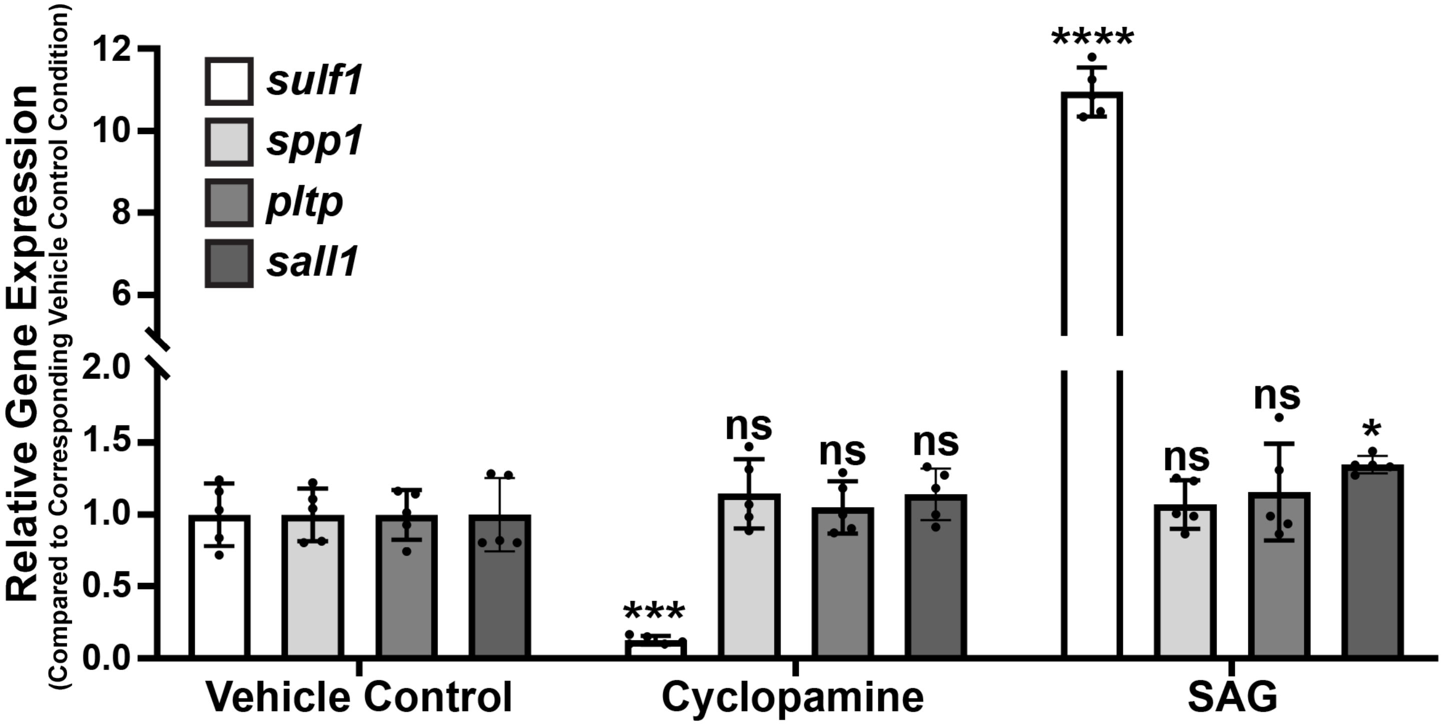

Supplementary Figure 9. Validation of *L. lugubris* fibroblast cell isolation pools via MACS® bead depletion. (a-f) Representative FISH of *L. lugubris* lizard tail fibroblast cell isolation pools **(a-c)** before and **(d-f)** after treatment with MACS® beads, analyzed for FCTCs (*col3a1*), endothelial cells (*vwf*), muscle-related cells (*ckm*) and immune cells (*aoah*). Bar = 25 µm. **(g)** Quantification of FISH percentage area of respective cell types before and after MACS® bead depletion treatment in fibroblast cultures. n = 10 fibroblast cell pools quantified each before and after treatment. Paired two-way *t*-tests were used between respective cell populations. ****, $p <$ 0.0001. Data are presented as mean values +/- standard deviation. Source data are provided as a Source Data file.

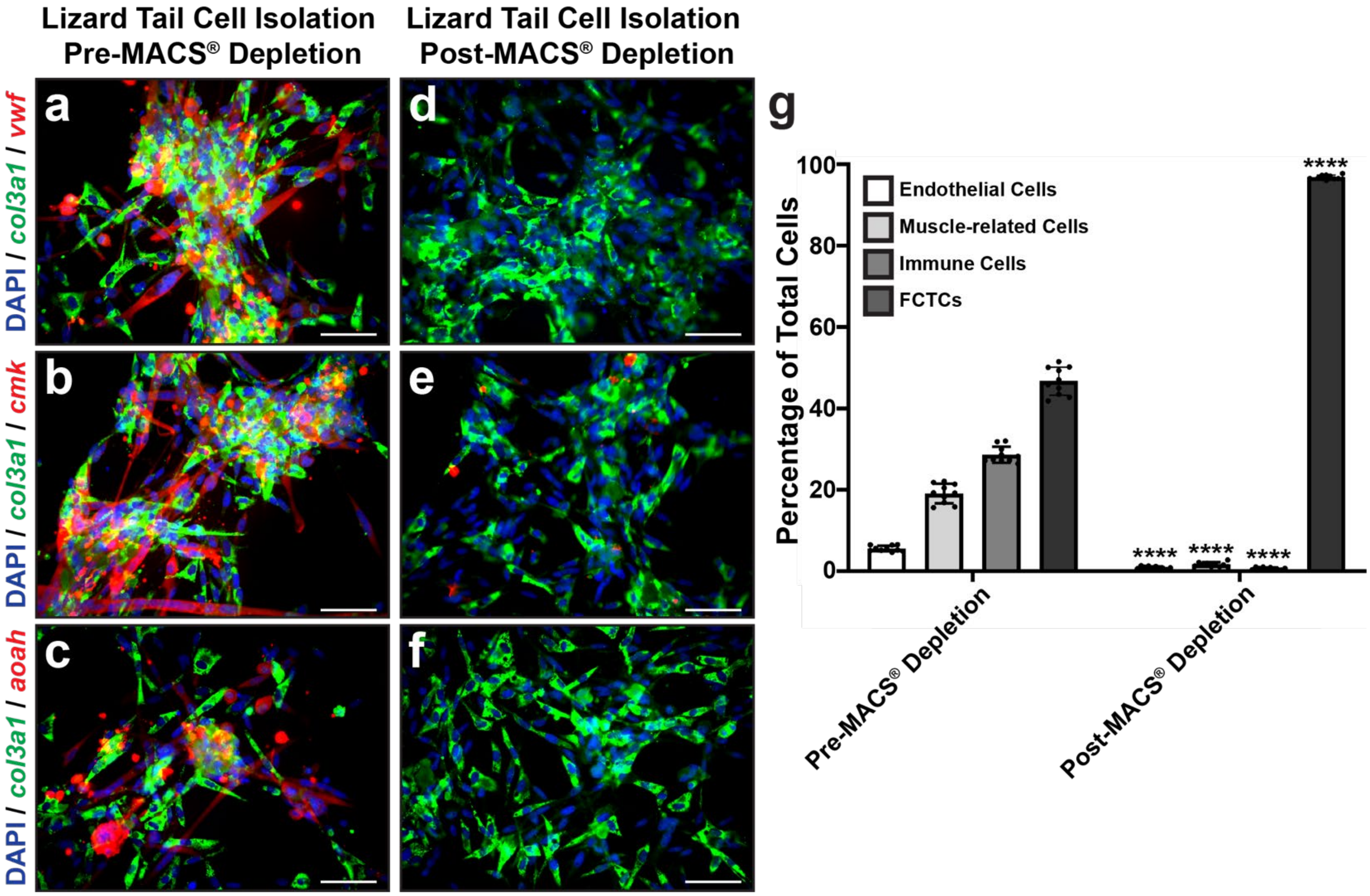

Supplementary Figure 10. Regenerating lizard tail skeletons exhibit proximodistal gradients of *sulf1* and *sox9*. Representative sagittal sections of tails collected 14 DPA from lizards (*A. carolinensis*) treated with **(a, b)** vehicle control, **(c, d)** cyclopamine, or **(e, f)** SAG, and analyzed by histology/ISH for *sulf1* and *sox9*. n = 10 lizards per treatment condition. et, ependymal tube; ve, vertebra. Bar = 500 μm.

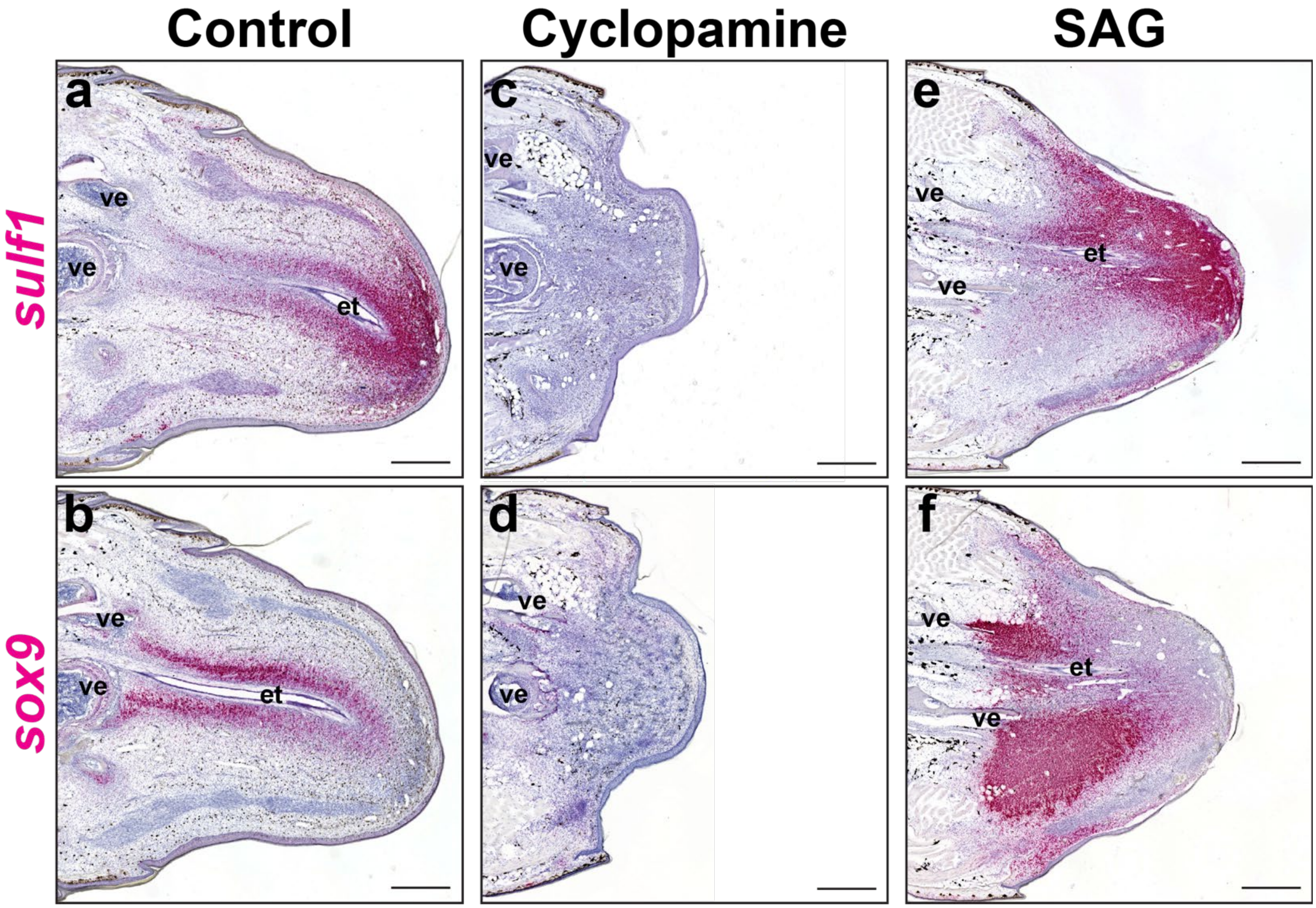

Supplementary Figure 11. Comparing chondrogenic abilities of tail and limb fibroblasts *in vivo*. (a) Experimental scheme for *L. lugubris* tail blastema and amputated limb fibroblast transplantations. (1) FCTCs are isolated from amputated original (0 DPA) tails and left hind limbs of donor lizards. (2) Tail FCTCs are labeled with DiI, while limb FCTCs are labeled with DiO. (3) Labeled FCTCs are co-transplanted into freshly amputated tail stumps (0 DPA) of SAG-treated recipient lizards. (4) Following 14 and 28 days of SAG treatment, regenerated tails are analyzed via Col2 immunofluorescence staining (IF), *sulf1* FISH, and fluorescence microscopy. **(b-i)** Representative fluorescent and histological analysis of tails regenerated by lizards co-injected with DiI-labeled tail FCTCs and DiO-labeled limb FCTCs, analyzed by **(b-e)** *sulf1* FISH 14 days post-transplantation (DPT)/14 DPA and **(f-i)** Col2 IF 28 DPT/28 DPA. DiI, DiO, and *sulf1* or Col2 signals are presented separately and together to highlight co-localization or lack thereof. Green arrowheads denote DiO^{+} cells and red arrowheads mark DiI^{+} cells. Bar = 50 µm. **(j, k)** Quantification of DiI- and DiO-labeled cells **(j)** co-expressing *sulf1* 14 DPT and **(k)** incorporated within Col2^{+} cartilage regions 28 DPT. n = 50 cell counts measured from 5 images among 10 different animals/tails for each condition. Unpaired two-way *t*-tests with Welch's correction for unequal variances were used. ns, not significant. Data are presented as mean values +/- standard deviation. Source data are provided as a Source Data file.

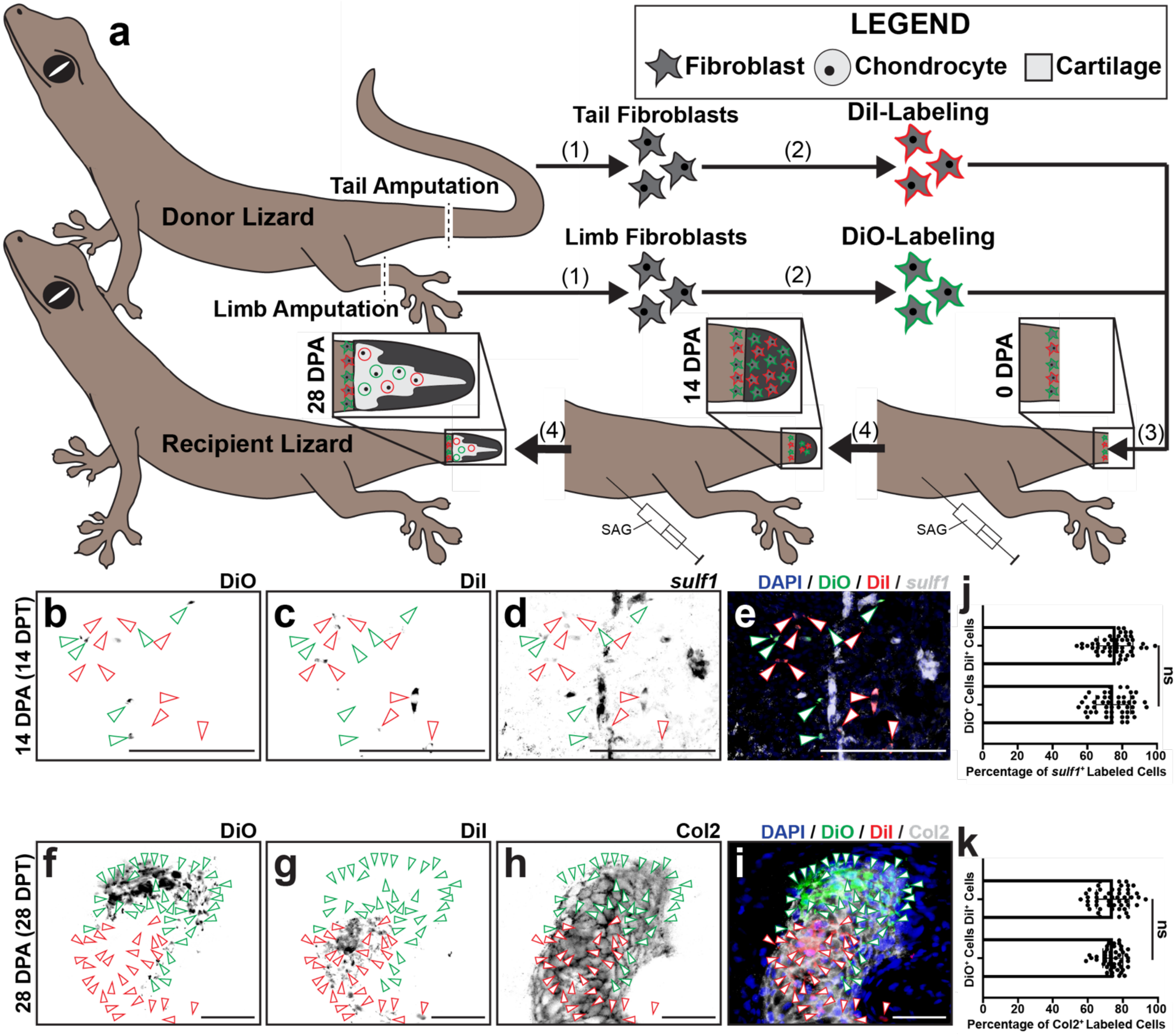

Supplementary Figure 12. Comparing chondrogenic abilities of *sulf1*⁺ tail blastema cells and *sulf1*⁻ tail fibroblasts *in vivo,* separately, in SAG- and vehicle control-treated recipient lizards. (a-l) Representative histological analysis of tails regenerated by **(a-c, g-i)** PBS vehicle control- or **(d-f, j-l)** SAG-treated recipient lizards pre-injected with **(a-f)** DiI-labeled blastema (14 DPA) tail-derived FCTCs or **(g-l)** 14 DPA limb-derived FCTCs and analyzed by Col2 IF and fluorescence microscopy 14 DPT/28 DPA. DiI and Col2 signals are presented separately and together to highlight co-localization or lack thereof. Green arrowheads mark DiI^+ cells. Bar = 50 μm. **(m)** Quantification of DiI-labeled cells incorporated within $Col2^+$ cartilage regions 14 DPT. n = 50 cell counts measured from 5 images among 10 different animals/tails for each condition. Two-way ANOVA with pairwise Tukey's adjustment for multiple comparisons was used. ****, adjusted $p < 0.0001$; ns, not significant (adjusted $p = 0.7939$, Tukey's). Data are presented as mean values +/- standard deviation. Source data are provided as a Source Data file.

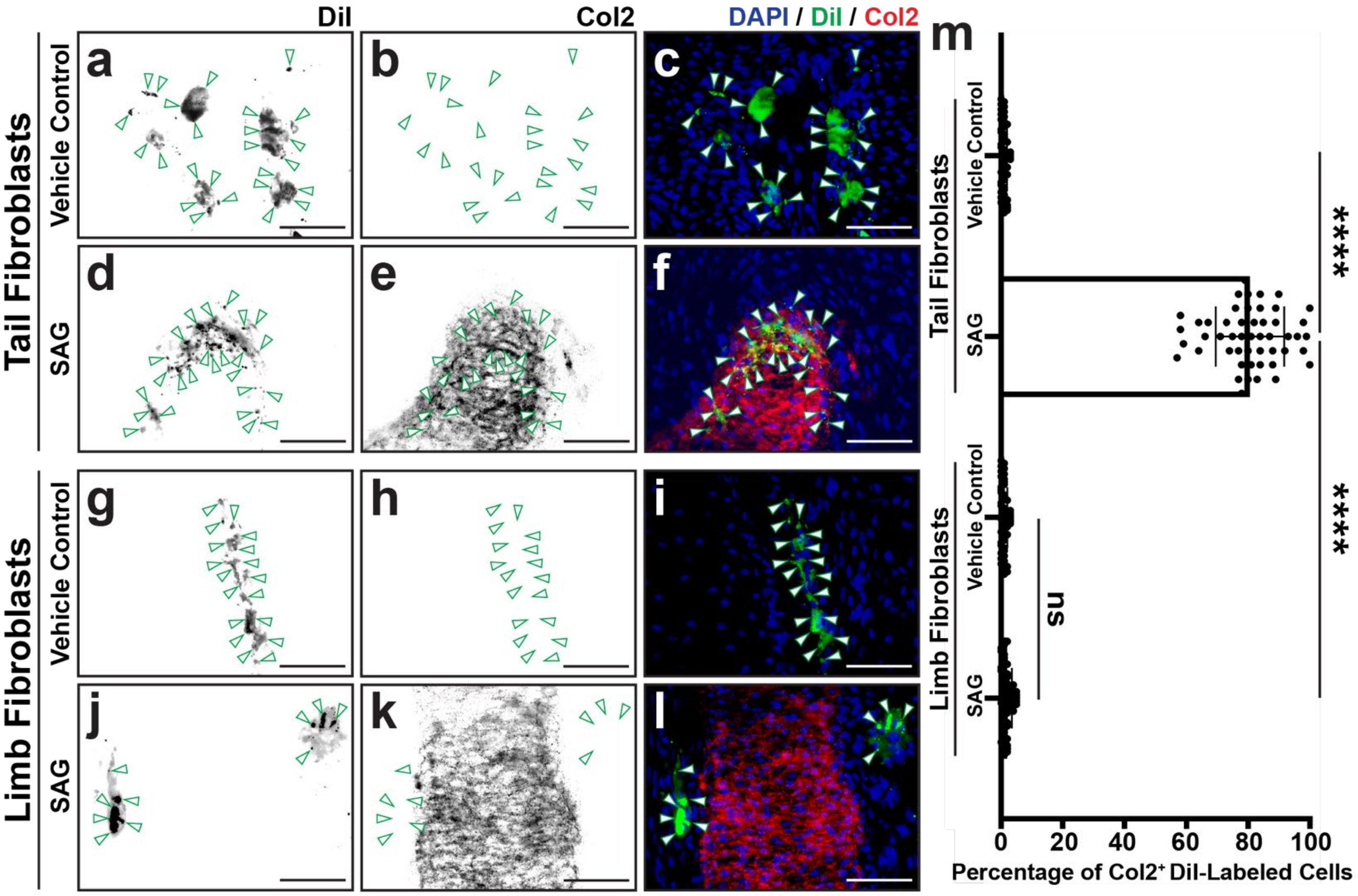

Supplementary Figure 13. Regenerated lizard tails exhibit distinct macrophage, osteoclast, and septoclast-like cell populations. (a-c) SPRING[3] visualization of regenerating lizard (*A. carolinensis*) tails highlighting distinct macrophage, osteoclast, and septoclast-like cell populations (herein, septoclasts). *Ctsb*$^{+}$ *ctsk*$^{+}$ osteoclasts clustered with *ctsb*$^{+}$ *ctsk*$^{-}$ macrophages, while *ctsb*$^{-}$ *ctsk*$^{+}$ septoclasts clustered with *col4a1*$^{+}$ pericytes. (**d**) Lizard tail blastema (14 DPA) analyzed with *col4a1* and *vwf* (endothelial cells) FISH toward validating *col4a1* as a pericyte marker in regenerating lizard tails. *Col4a1*$^{+}$ pericytes associate with *vwf*$^{+}$ blood vessels. n = 3 lizards for representative FISH. bv, blood vessel. Bar = 100 μm.

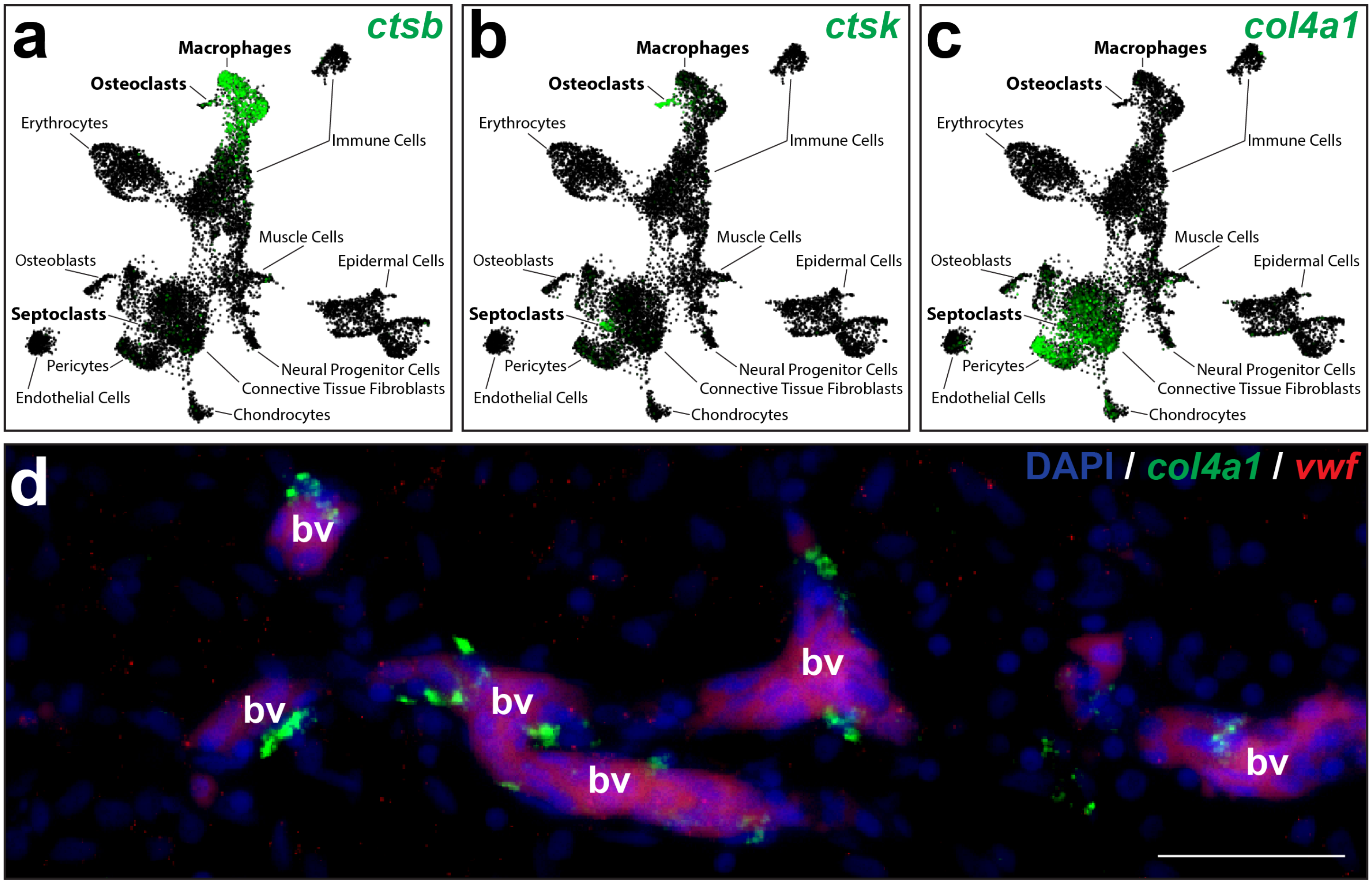

Supplementary Figure 14. Phagocytic macrophage and osteoclast populations peak during inflammatory stage 7 DPA. (a-e) Representative histological and FISH analysis of phagocytic macrophages and osteoclasts (*ctsb*) and immune cells (*aoah*) in lizard (*A. carolinensis)* tails from 0-14 DPA. Bar = 500 µm. **(f)** Quantification of positive FISH percentage area for each cell population over total regenerated tail sample area per time point. Abundant immune cells and phagocytic macrophages and osteoclasts are observed 1-7 DPA, characteristic of the inflammatory stage of tail regeneration, with phagocytic cells peaking 7 DPA**.** n = 5 images quantified from 1 image each among 5 different animals/tails per time point. Data are presented as mean values +/- standard deviation. Source data are provided as a Source Data file.

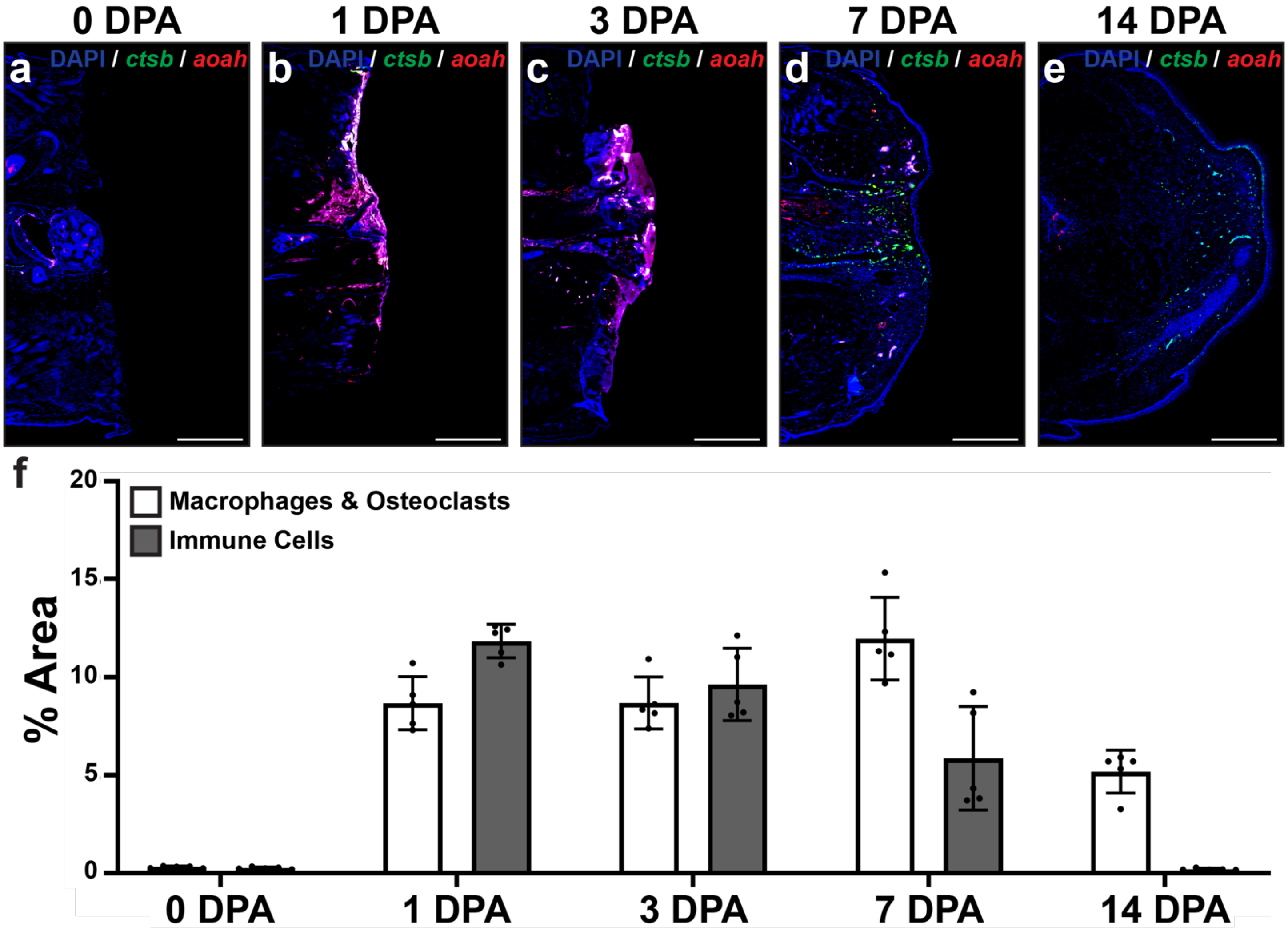

Supplementary Figure 15. Clodronate treatment depletes phagocyte populations, prevents blastema formation, and inhibits blastema fibroblast gene expression changes. Representative sagittal sections of lizard (*A. carolinensis*) tails collected 14 DPA, pre-treated with **(a-e)** PBS liposomes or **(f-j)** clodronate liposomes, and analyzed by **(a, b, f, g)** histology/ISH for *sulf1* and *spp1* and by **(c-e, h-j)** FISH for *ctsb, ctsk*, and *col4a1*. **(d, e, i, j)** Higher magnification views of corresponding regions identified in **(c)** and **(h)** highlighting *ctsb*$^{+}$ *ctsk*$^{-}$ *col4a1*$^{-}$ macrophages (m, arrowhead), *ctsb*$^{+}$ *ctsk*$^{+}$ *col4a1*$^{-}$ osteoclasts (o, arrowhead), and *ctsb*$^{-}$ *ctsk*$^{+}$ *col4a1*$^{+}$ septoclasts (s, arrowhead). n = 12 lizards per treatment. et, ependymal tube; sc, spinal cord; ve, vertebra. Bar = 50 µm.

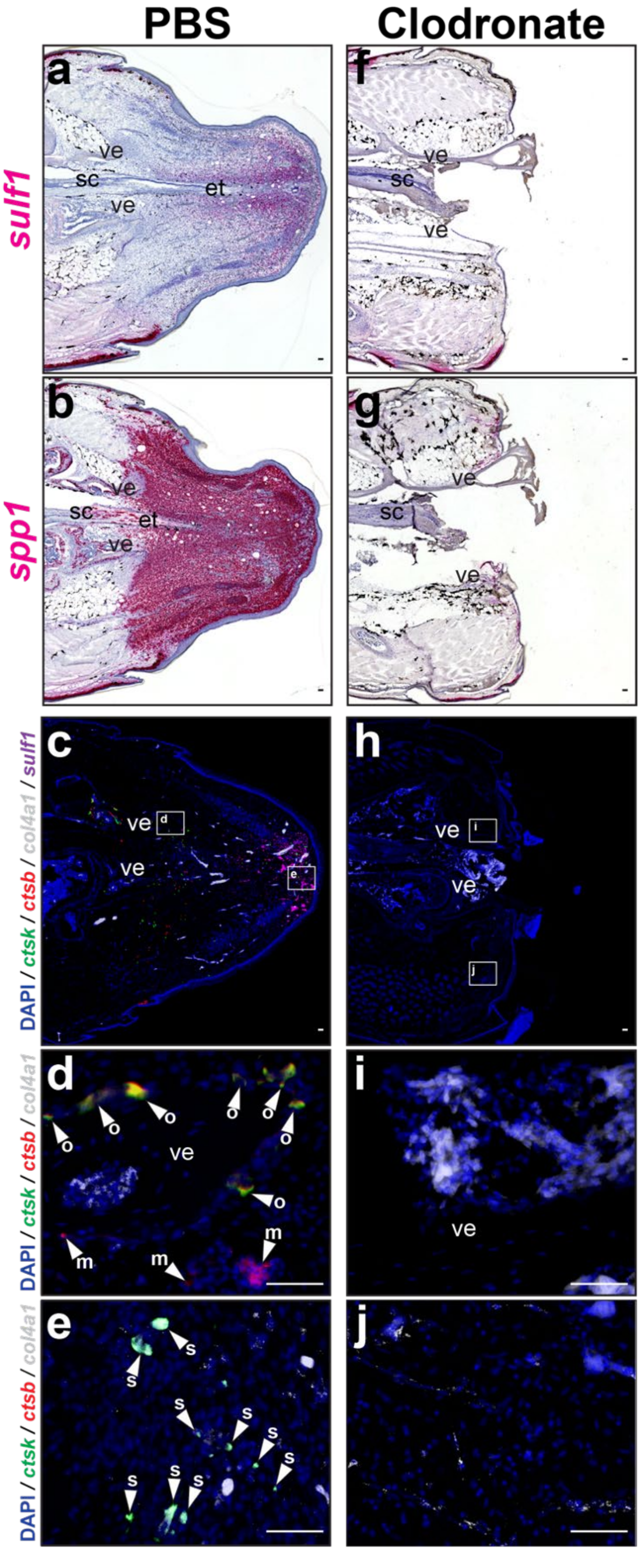

Supplementary Figure 16. *Sulf1* and *spp1* expression co-localize in lizard blastema fibroblasts. Representative sagittal sections of lizard *(A. carolinensis)* tails **(a)** 7 DPA in the inflammatory stage and **(b)** 14 DPA in the blastema stage, analyzed via histology/FISH for *sulf1* and *spp1*. **(a)** *Spp1* expression, but not *sulf1* expression, is observed in inflammatory stage fibroblasts. **(b)** *Sulf1* and *spp1* are co-expressed in distal blastema stage fibroblasts. n = 3 tail samples per time point. Bar = 500 µm.

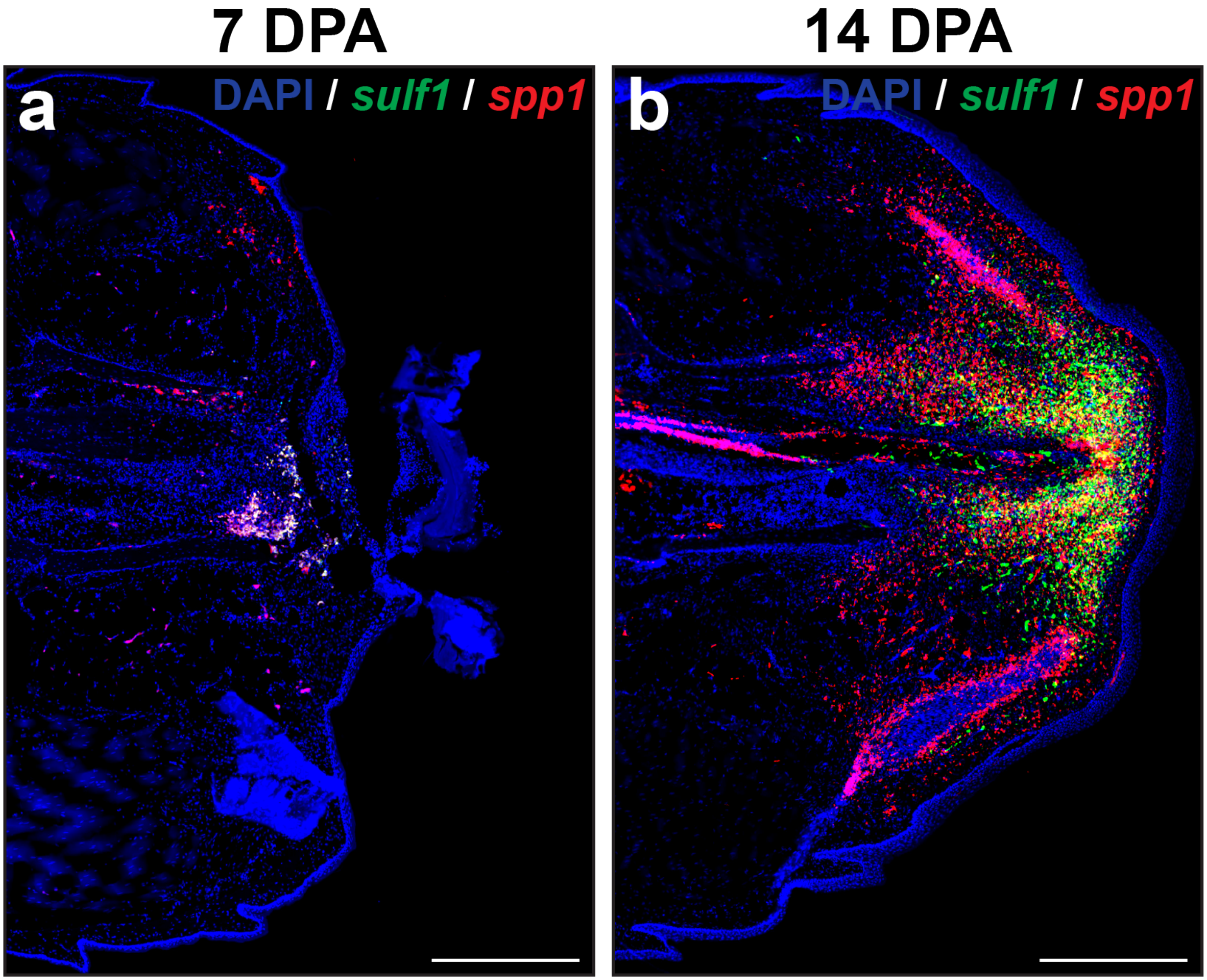

Supplementary Figure 17. Clodronate liposome treatments deplete phagocytes and inhibits tail regeneration in both *A. carolinensis* and *L. lugubris* lizards. (a-h) Gross morphology of **(a, b)** green anole (*A. carolinensis*) and **(e, f)** mourning gecko (*L. lugubris*) treated with DiI and PBS or clodronate liposomes, assessed for regenerated tail length 21 DPA. Dashed lines mark amputation planes. **(c, d, g, h)** Fluorescence microscopy following Fluoroliposome® DiI injection, a dye that fluoresces when engulfed by phagocytic cells[2], in 21 DPA tails from **(c, d)** *A. carolinensis* and **(g, h)** *L. lugubris.* Bar = 1 mm. **(i, j)** Quantification of **(i)** regenerated tail length and **(j)** phagocytic cell density in *A. carolinensis* and *L. lugubris* with PBS or clodronate liposome treatment. n = 10 samples/images quantified per species and treatment for tail length and phagocytic cell density. Unpaired two-way *t*-tests with Welch's correction for unequal variances were used. ****, $p < 0.0001$. Data are presented as mean values +/- standard deviation. Source data are provided as a Source Data file.

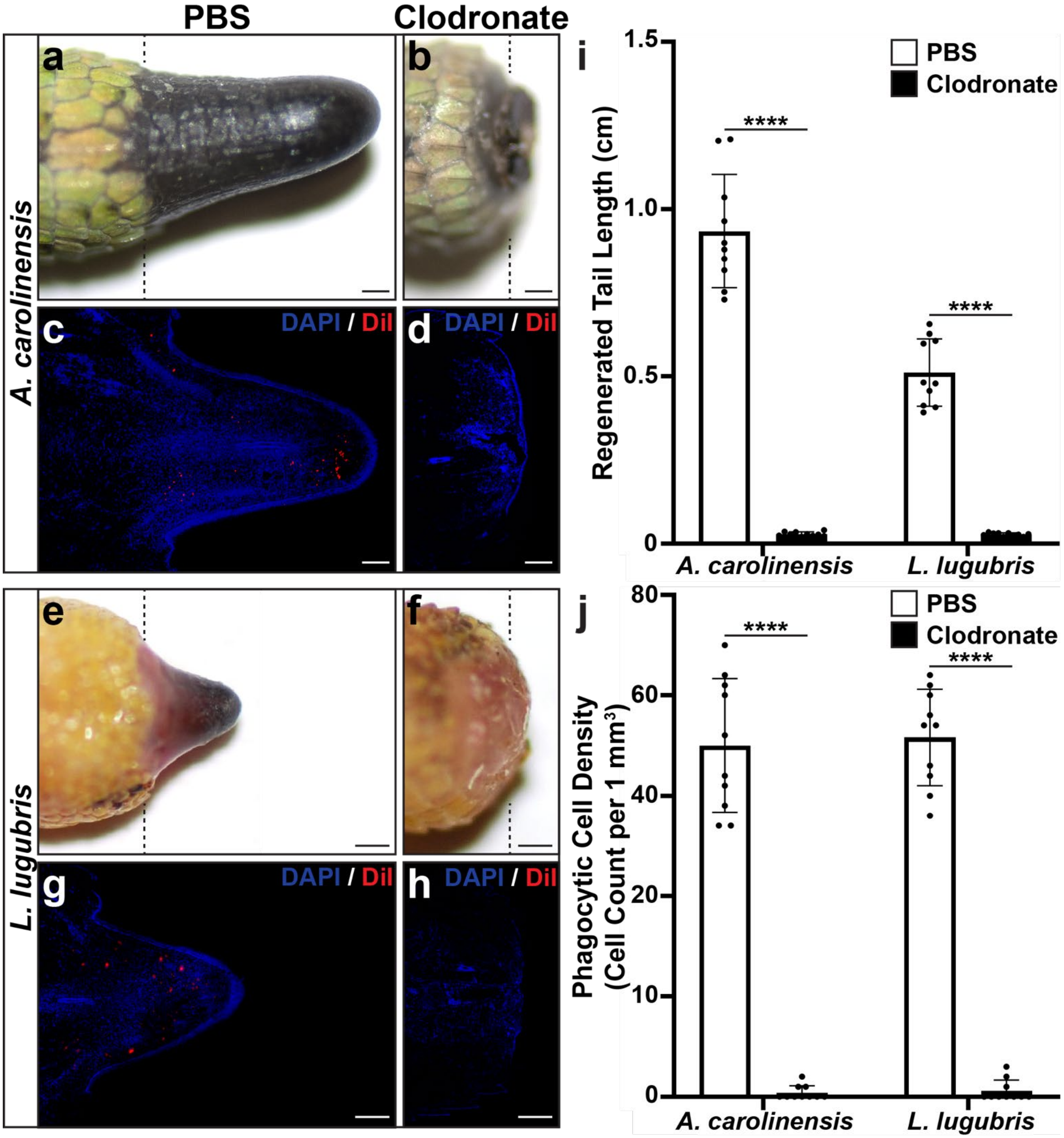

Supplementary Figure 18. Comparing chondrogenic abilities of tail blastema fibroblasts with and without phagocytic cell exposure *in vivo*, separately, in SAG- and vehicle control-treated recipient lizards. (a-l) Representative histological analysis of tails regenerated by **(a-c, g-i)** PBS vehicle control- or **(d-f, j-l)** SAG-treated recipient lizards pre-injected with DiI-labeled blastema (14 DPA) tail-derived fibroblasts from donor lizards treated with **(a-f)** PBS liposomes or **(g-l)** clodronate liposomes and analyzed by Col2 IF and fluorescence microscopy 14 DPT/28 DPA. DiI and Col2 signals are presented separately and together to highlight co-localization or lack thereof. Green arrowheads mark DiI^{+} cells. Bar = 50 μm. **(m)** Quantification of DiI-labeled cells incorporated within Col2^{+} cartilage regions 14 DPT. n = 50 cell counts measured from 5 images among 10 different animals/tails for each condition. Two-way ANOVA with pairwise Tukey's adjustment for multiple comparisons was used. ****, adjusted $p < 0.0001$; ns, not significant (adjusted $p = 0.8478$, Tukey's). Data are presented as mean values +/- standard deviation. Source data are provided as a Source Data file.

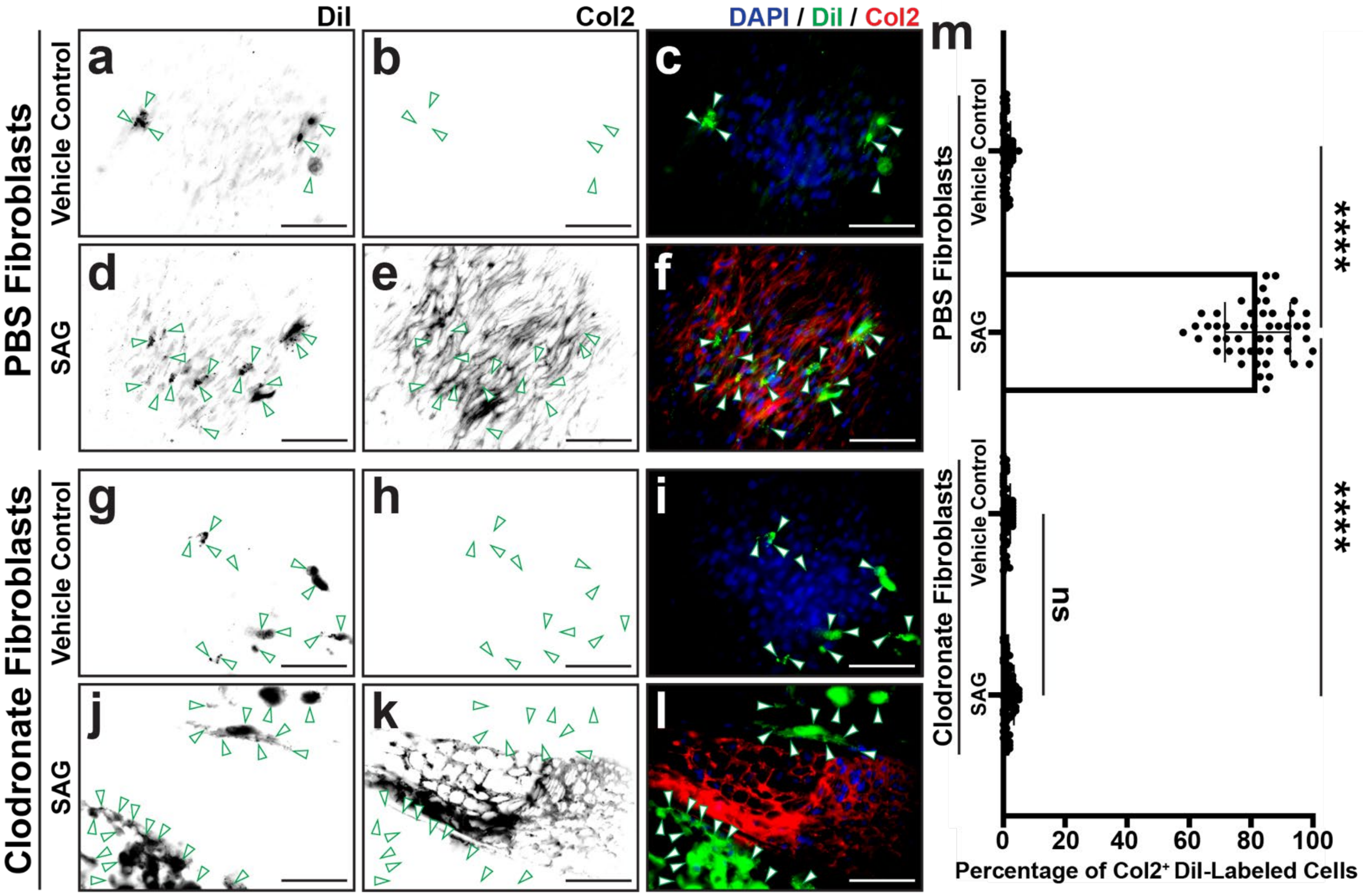

Supplementary Figure 19. Validation of bone marrow cell-derived macrophages and tail vessel pericyte-derived septoclasts differentiated in culture. (a, b) Phagocytosis assays of **(a)** bone marrow-derived macrophages and **(b)** tail blood vessel-derived septoclasts incubated with 0, 1 or 100 µg/mL pHrodo™ green *E. coli* bioparticles. Percentages indicate quantity of phagocytic cells. **(c, g)** Overlaid brightfield and green fluorescence micrographs indicating phagocytic cells within both **(c)** macrophage and **(g)** septoclast cell cultures. **(d-f, h-j)** Representative fluorescence microscopy of **(d-f)** macrophage and **(h-j)** septoclast cell cultures, analyzed by *itgam/cd11b, ctsb*, and *col4a1* FISH and Ctsk IF. Both macrophages and septoclasts expressed phagocyte marker *itgam/cd11b*. Only macrophages expressed *ctsb*, while only septoclasts exhibited *col4a1* expression and Ctsk^{+} intracellular vesicles (arrowheads). Bar = 50 µm. n = 5 isolated macrophage and septoclast cell pools stained each. **(k, l, n, o)** Flow cytometric analysis of cultures of **(k, l)** macrophage and **(n, o)** septoclasts labeled with **(l, o)** anti-Ctsb and anti-Ctsk primary antibodies with indicated fluorescent tags. **(k, n)** Control **(k)** macrophage and **(n)** septoclasts incubated with isotype controls for primary antibodies with matching fluorescent tags to define quadrant gating. Solid lines within plots denote gating boundaries. **(m, p)** Quantification of flow cytometry quadrants in **(l, n)**, respectively. n = 5 isolated macrophage and septoclast cell pools stained and analyzed by separate flow cytometry experiments. Cell populations within quadrant are indicated as percentages. One-way Welch's ANOVA for unequal variances with Dunnett's T3 multiple comparisons tests were used. ****, adjusted $p < 0.0001$, compared to all other quadrants (Dunnett's T3). Data are presented as mean values +/- standard deviation. Source data are provided as a Source Data file.

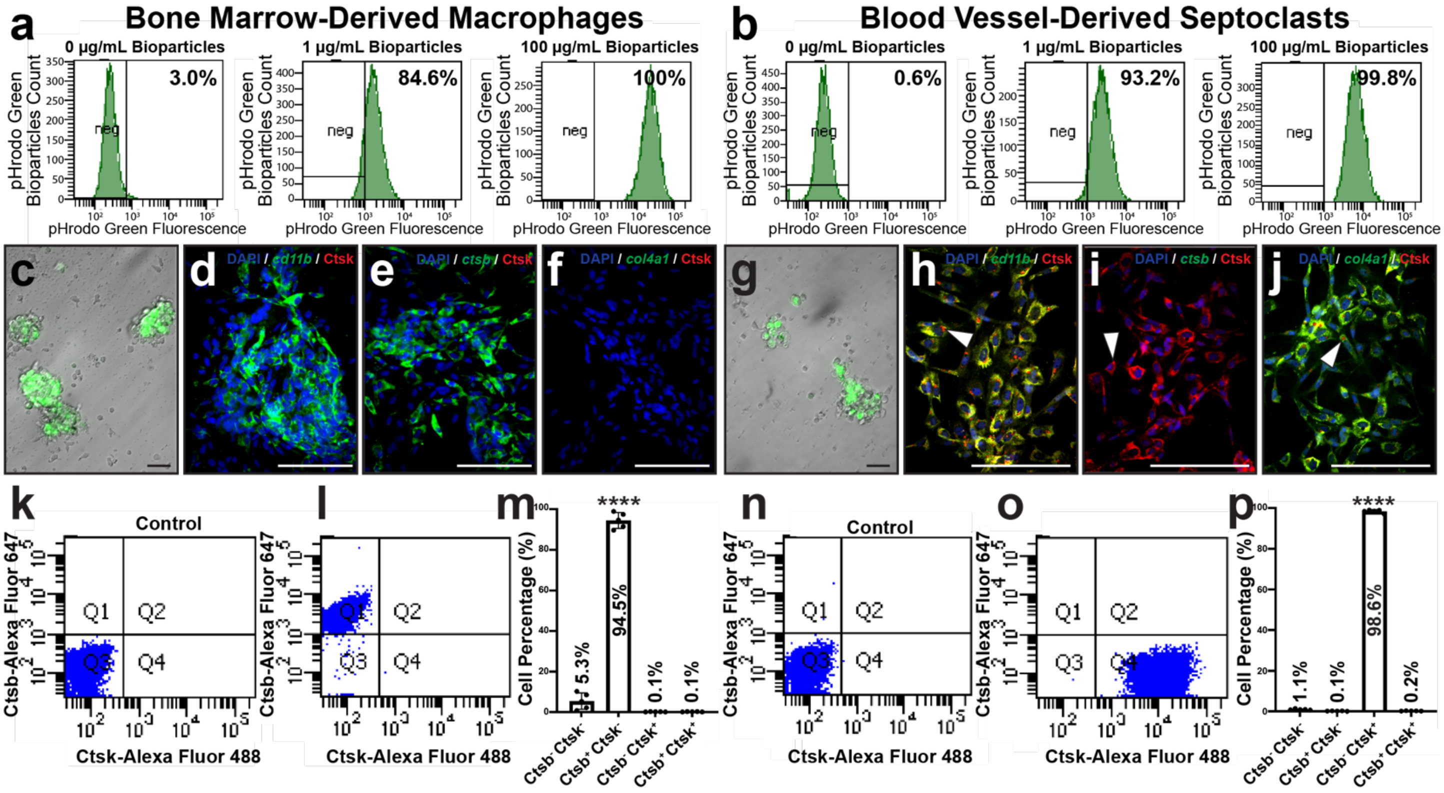

Supplementary Figure 20. Septoclast cell-conditioned media does not induce amputated tail fibroblast *sulf1* expression in the absence of endogenous phagocyte populations and Hedgehog signaling. (a) Experimental scheme for testing effects of biomolecules secreted by macrophage and septoclast populations on FCTC marker gene expression in clodronate liposome-treated lizard (*A. carolinensis*) tails. (1) Bone marrow and tail blood vessel cells are isolated and (2) differentiated into macrophages and septoclasts *in vitro*, respectively. (3) Macrophage- and septoclast-conditioned media (M-CM and S-CM) are collected, concentrated, and embedded in alginate beads, together and separately. (4) Alginate beads are implanted into amputated tails (0 DPA) of lizards co-treated with clodronate liposomes and PBS (vehicle control for SAG treatment). **(b-q)** Representative sagittal sections of lizard tails 14 DPA co-treated with PBS and clodronate liposomes, implanted with M-CM and/or S-CM beads, and analyzed via histology/ISH/FISH for *sulf1, spp1, ctsk, ctsb*, and *col4a1* expression. Higher magnification fluorescent views of indicated regions around **(d, h, l, p)** vertebra and **(e, i, m, q)** implanted bead sites in **(b, c, f, g, j, k, n, o)**. n = 8 lizards/samples per treatment condition. *, location of implanted bead; ve, vertebra. Bar = 50 µm.

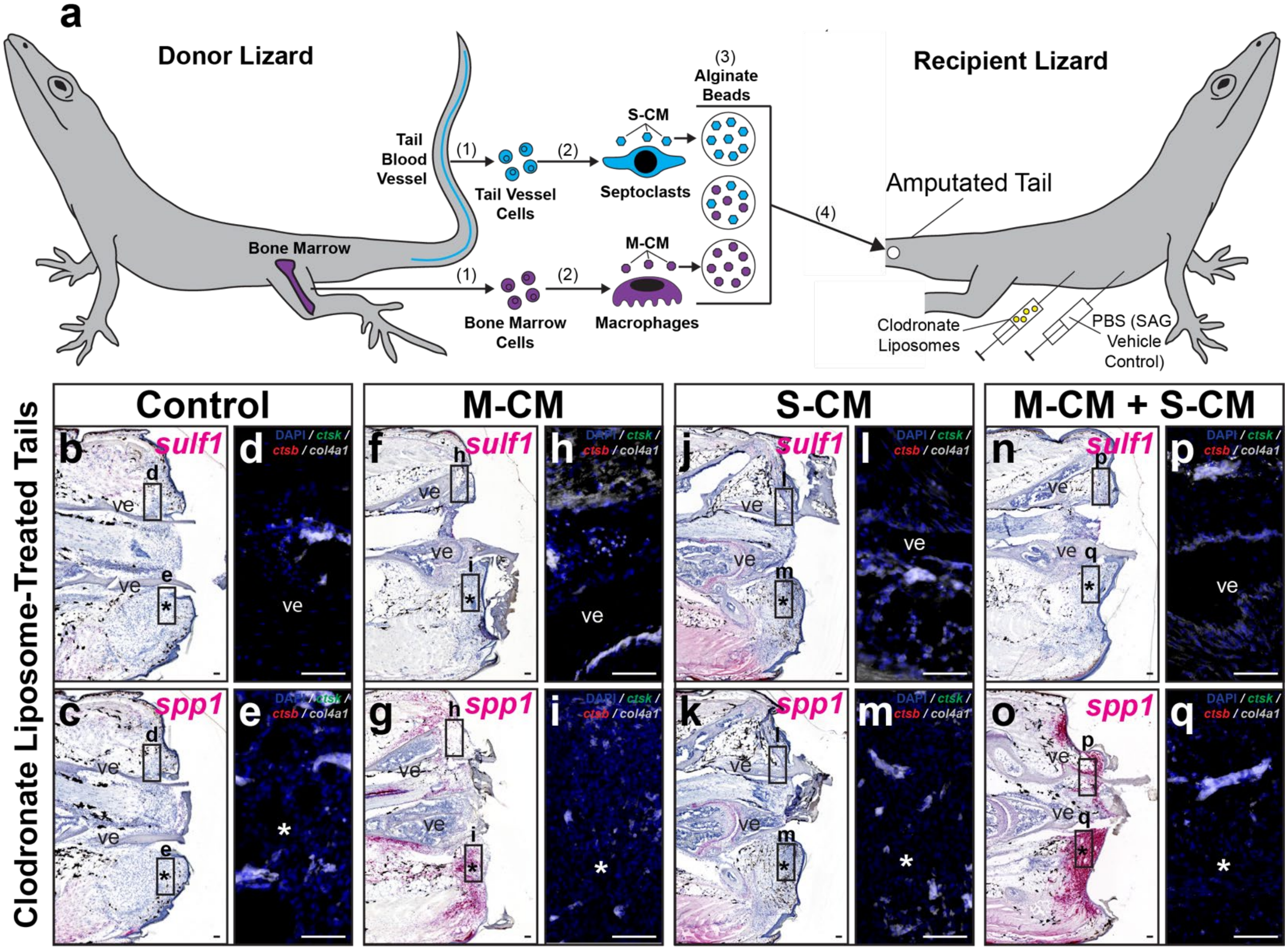

Supplementary Figure 21. Unconditioned phagocyte media does not induce *sulf1, spp1* or *sox9* expression in clodronate liposome-treated tails or untreated amputated limbs. (a-d) Representative sagittal sections of lizard (*A. carolinensis*) tails 14 DPA co-treated with clodronate liposomes and **(a, b)** vehicle control (PBS) or **(c, d)** SAG, implanted with beads impregnated with unconditioned macrophage/septoclast media, and analyzed via histology/ISH for *sulf1* and *spp1* expression. **(e-j)** Representative sagittal sections of lizard limbs 28 DPA treated with **(e-g)** vehicle control or **(h-j)** SAG, implanted with unconditioned macrophage/septoclast media-soaked beads, and analyzed via histology/ISH for *sulf1, sox9*, and *spp1* expression. n = 8 lizards/samples per treatment condition. *, location of implanted bead; tb, tibia; ve, vertebra. Bar = 50 µm.

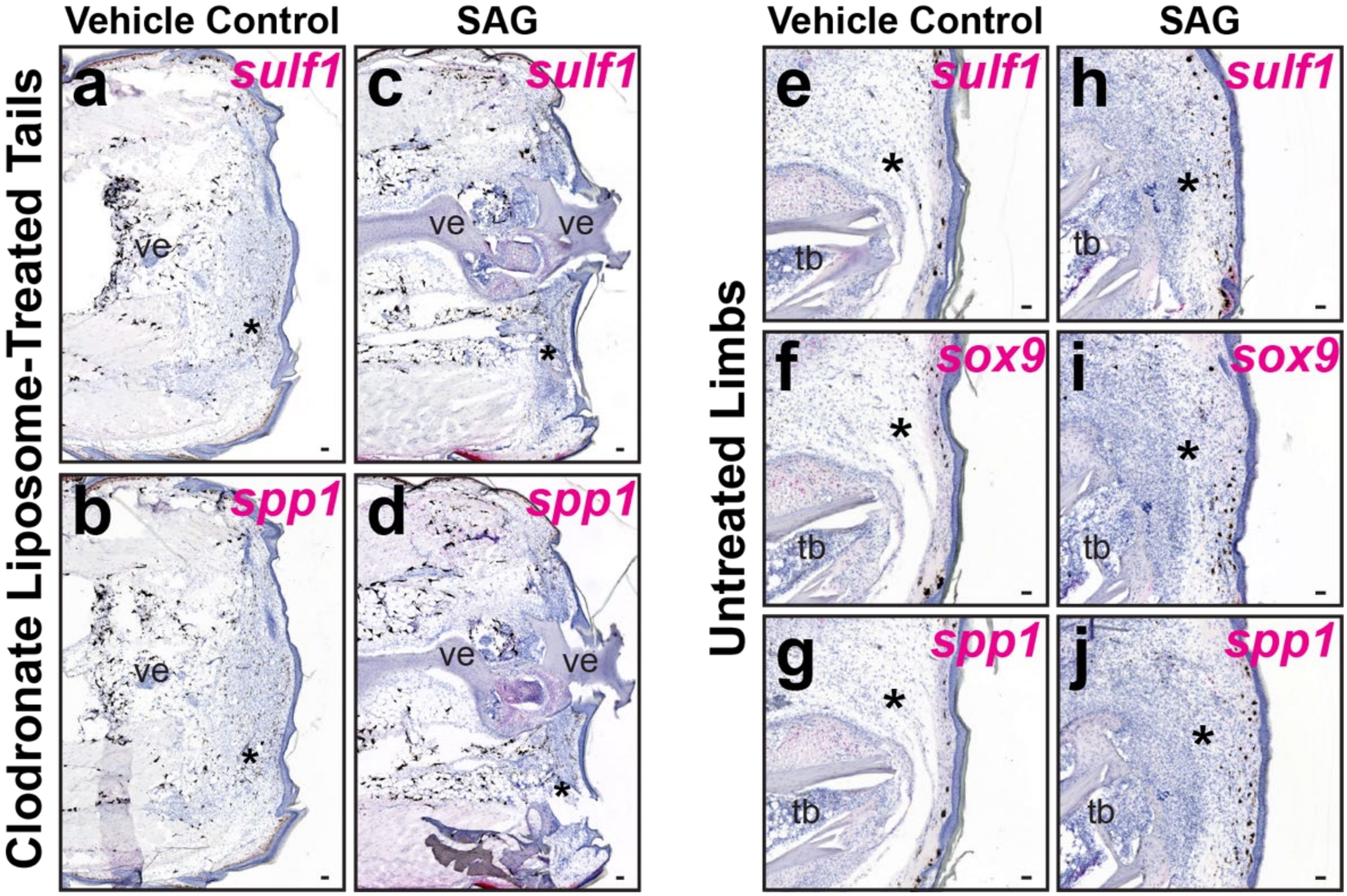

Supplementary Figure 22. Septoclast cell-conditioned media does not induce amputated tail fibroblast *sulf1* or *sox9* expression in the absence of Hedgehog signaling. (a) Experimental scheme for testing effects of macrophage- and septoclast-conditioned media on FCTC marker gene expression in lizard (*A. carolinensis*) limbs. (1) Bone marrow and tail blood vessel cells are isolated and (2) used to derive macrophages and septoclasts *in vitro*, respectively. (3) Media conditioned by macrophages and septoclasts (M-CM and S-CM) are collected, concentrated, and embedded in alginate beads, together and separately. (4) Alginate beads are implanted into amputated limbs of PBS-treated (vehicle control of SAG treatment) lizards 7 DPA. **(b-u)** Representative sagittal sections of lizard limbs 28 DPA treated with vehicle control, implanted with M-CM and/or S-CM beads, and analyzed via histology/ISH/FISH for *sulf1, sox9, spp1, ctsk, ctsb*, and *col4a1* expression. Higher magnification fluorescent views of indicated regions in **(b-d, g-i, l-n, q-s)** depict indicated regions around **(e, j, o, t)** implanted bead and **(f, k, p, u)** tibia. n = 8 lizards/samples per treatment condition. *, location of implanted bead; m (arrowhead), macrophage; o (arrowhead), osteoclast; tb, tibia. Bar = 50 µm.

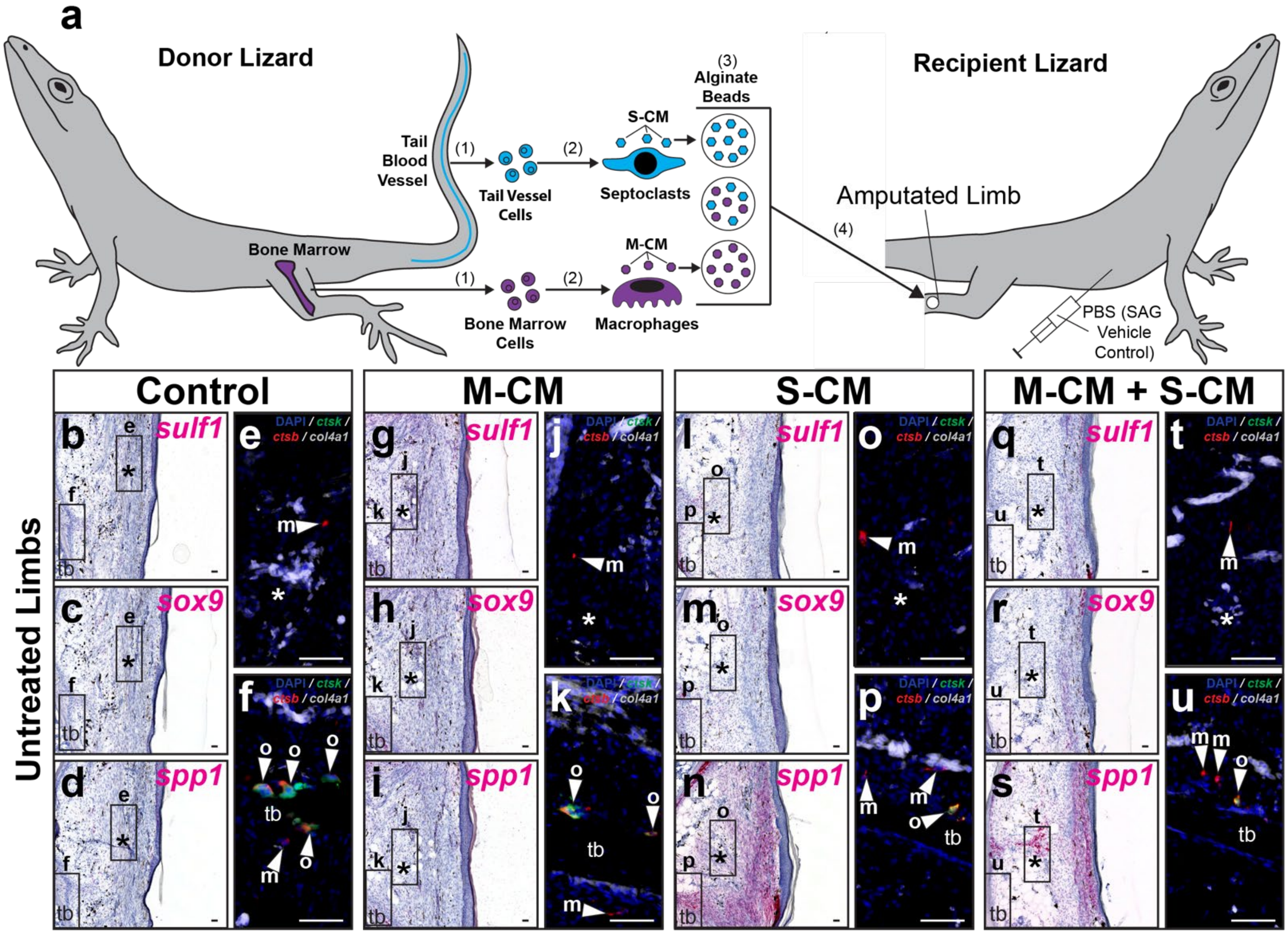

Supplementary Figure 23. Gating strategy for flow cytometry analysis in Supplementary Figure 19. (a, b) Gating strategy for flow cytometry analysis of phagocytosis assays of **(a)** bone marrow-derived macrophages (Supplementary Fig. 19a) and **(b)** tail blood vessel-derived septoclasts (Supplementary Fig. 19b) incubated with pHrodo™ green *E. coli* bioparticles. Cells were single-gated from total events (black) based on forward scatter (FSC) and side scatter (SSC). Double FSC and SSC gates (green) were used to select green-fluorescing positive cells for analysis. **(c, d)** Gating strategy for flow cytometry analysis of **(c)** macrophage (Supplementary Fig. 19l) and **(d)** septoclasts (Supplementary Fig. 19o), incubated with fluorescent-conjugated primary antibodies anti-Ctsb-Alexa Fluor™ 647 and anti-Ctsk-Alexa Fluor™ 488. Cells were single-gated from total events (black) based on FSC and SSC. Double FSC and SSC gates (blue) were used to select cells for analysis.

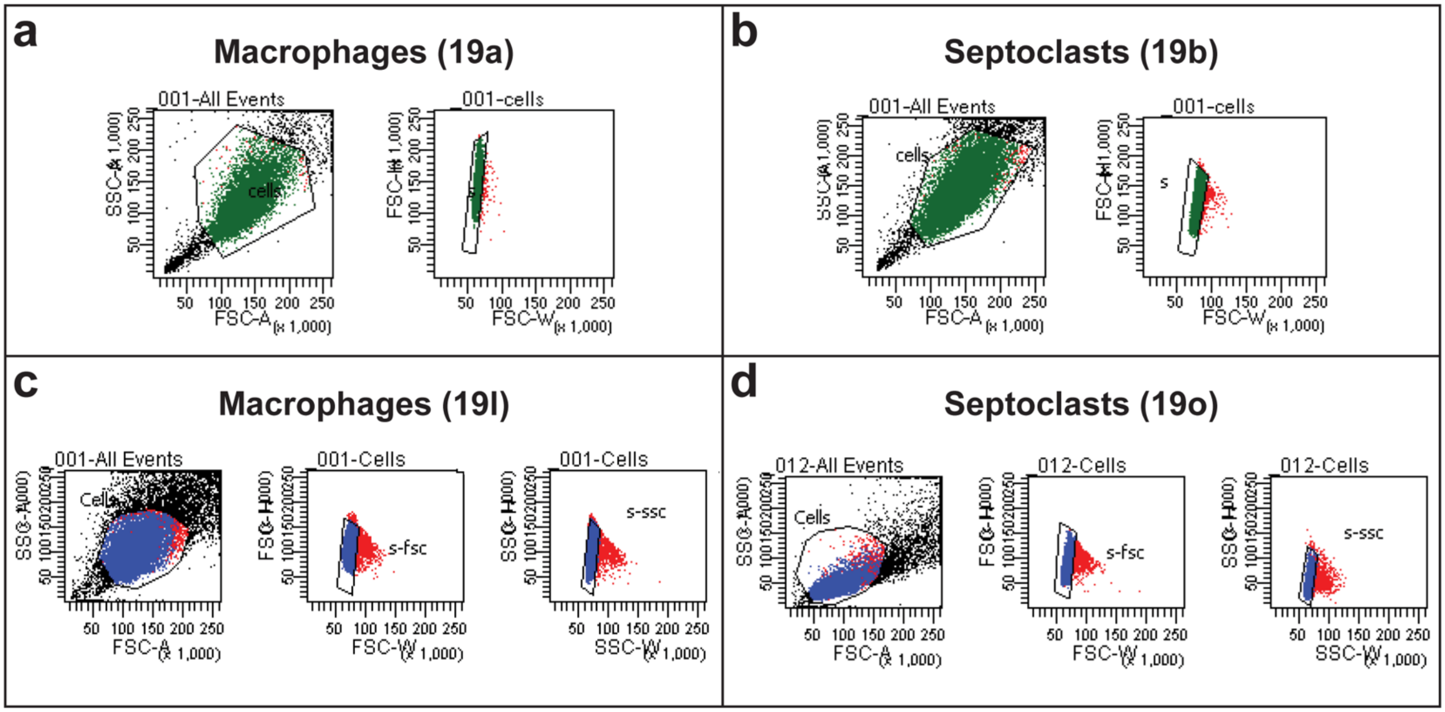

SUPPLEMENTARY TABLES

Supplementary Table 1. Relative fibroblast marker gene expression throughout tail regeneration. Relative gene expression levels of FCTC markers (assessed from ISH in Figure 2 & Supplementary Figure 5). -, no detectable expression; +, low expression; +++, high expression.

Marker gene	Homeostatic Fibroblasts (0 DPA)	Injury Fibroblasts (7 DPA)	Blastema Fibroblasts (14 DPA)	Chondrocytes (28 DPA)
cdh11	+	+	+++	-
col3a1	+++	+++	+++	+++
col12a1	-	+	+++	+++
mdk	-	+	+++	+
pltp	-	-	+++	+
sall1	-	-	+++	+
sox9	-	-	+++	+++
sparc	-	+	+++	+++
spp1	-	+++	+++	-
sulf1	-	+	+++	+
tnl	-	+++	+++	-

Supplementary Table 2. Catalog information for commercial probes critical to *in situ* hybridization (ISH) and fluorescent ISH experimental outcomes. All probes were custom designed by supplier Advanced Cell Diagnostics, Inc. to species-specific mRNA sequences for respective genes.

**Reagent**	**Catalog Number**
RNAscope™ Probe-Acar-aoah	896491
RNAscope™ Probe-Acar-cdh11	896421
RNAscope™ Probe-Acar-LOC100562721 (*ckm)*	882301
RNAscope™ Probe-Acar-col1a1	1154551
RNAscope™ Probe-Acar-col2a1	882331
RNAscope™ Probe-Acar-col3a1	896371
RNAscope™ Probe-Acar-col4a1	896381
RNAscope™ Probe-Acar-col12a1	896401
RNAscope™ Probe-Acar-ctsb	896341
RNAscope™ Probe-Acar-LOC100561728 (*ctsk*)	882321
RNAscope™ Probe-Acar-fabp7	896331
RNAscope™ Probe-Acar-gli1	1181451
RNAscope™ Probe-Acar-ifi30	896321
RNAscope™ Probe-Acar-itgam	896501
RNAscope™ Probe-Acar-LOC100566088 (*krt5*)	896481
RNAscope™ Probe-Acar-mdk	896231
RNAscope™ Probe-Acar-pltp	1058571
RNAscope™ Probe-Acar-sall1	1058561
RNAscope™ Probe-Acar-sox9	896451
RNAscope™ Probe-Acar-sparc	896441
RNAscope™ Probe-Acar-spp1	896251
RNAscope™ Probe-Acar-sulf1	1058551
RNAscope™ Probe-Acar-LOC100557189 (tenascin-like/*tnl*)	1058541
RNAscope™ Probe-Acar-vwf	896201
RNAscope™ Probe-Gj-sulf1	1123161

Supplementary Table 3. RT-PCR primer sequences. Forward and reverse primer sequences utilized in RT -PCR experiments by gene.

Gene	Forward Primer	Reverse Primer
gapdh	CCATGTTTGTGATGGGTGTC	CTATGGTGGTGAAGACGCCA
sulf1	GTTTGCCACGGGATTTCTGG	AGCTTCCTCCATCTTTACTTCCTG
spp1	TGTGTCTTCTGACCATCGCC	GGTGATGTGGGTGAGCATGA
pltp	TGTTCTTCCCTTTGCGGGAG	CCGAAGAACGAGGCTCTCAA
sall1	TGCATCGTCATCACCAGCTT	AACTCAAGTCCTCTGCAGGC

Supplementary Data 1. Statistical test reporting. ***Available as Excel data file.*** Legend: Summary of all statistical tests performed in Main and Supplementary Figures including statistical test with comparison (if applicable), test statistic, degrees of freedom, and p-values or adjusted p-values. W, one-way Welch's ANOVA test statistic (equivalent to ordinary ANOVA F); t, t-test and Dunnett's T3 multiple comparisons test statistic; F, two-way ANOVA test statistic; q, Tukey's multiple comparisons test statistic; $^*p$, adjusted p-value; DF, degrees of freedom; DFn, degrees of freedom (numerator); DFd, degrees of freedom (denominator); ns, not significant. Significance levels: *, $p < 0.05$; **, $p < 0.01$; ***, $p < 0.001$; ****, $p < 0.0001$.

See Supplementary Data 1 Excel data file.

SUPPLEMENTARY REFERENCES

1. ThermoFisher Scientific. pHrodo^{TM} Green *E. coli* BioParticles^{TM} Conjugate for Phagocytosis. https://www.thermofisher.com/order/catalog/product/P35366?SID=srch-srp-P35366.
2. ThermoFisher Scientific. *The Molecular Probes Handbook: A Guide to Fluorescent Probes and Labeling Technologies - 16.1 Probes for Following Receptor Binding and Phagocytosis*. (2010).
3. Weinreb, C., Wolock, S. & Klein, A. M. SPRING: a kinetic interface for visualizing high dimensional single-cell expression data. *Bioinformatics* **34**, 1246–1248 (2018).
