# Supplementary material for: Single-cell analysis of lizard blastema fibroblasts reveals phagocyte-dependent activation of Hedgehog-responsive chondrogenesis
Source: Nat Commun. 2023 Aug 10;14:4489. doi: 10.1038/s41467-023-40206-z (PMC10415409; doi:10.1038/s41467-023-40206-z)
Supplement: Supplementary file 3 — Description of Additional Supplementary Files [file 41467_2023_40206_MOESM3_ESM.pdf]

Description of Additional Supplementary Files

Supplementary Data 1. Statistical test reporting. Summary of all statistical tests performed in Main and Supplementary Figures including statistical test with comparison (if applicable), test statistic, degrees of freedom, and *p*-values or adjusted *p*-values. *W*, one-way Welch's ANOVA test statistic (equivalent to ordinary ANOVA *F*); *t*, *t*-test and Dunnett's T3 multiple comparisons test statistic; *F*, two-way ANOVA test statistic; *q*, Tukey's multiple comparisons test statistic; **p*, adjusted *p*-value; DF, degrees of freedom; DFn, degrees of freedom (numerator); DFd, degrees of freedom (denominator); ns, not significant. Significance levels: *, $p < 0.05$; **, $p < 0.01$; ***, $p < 0.001$; ****, $p < 0.0001$.
